# Beyond Perceptions: Meta-Analyses on Observed Parent–Child Interaction Behaviors and Childhood and Adolescent Depression

**DOI:** 10.1007/s10567-026-00572-8

**Published:** 2026-06-05

**Authors:** Wilma G. M. Wentholt, Marc L. Molendijk, Loes H. C. Janssen, Lisanne A. E. M. van Houtum, Marieke S. Tollenaar, Lenneke R. A. Alink, Bernet M. Elzinga

**Affiliations:** 1 Department of Clinical Psychology, Institute of Psychology, Leiden University, Leiden, The Netherlands; 2 Leiden Institute for Brain and Cognition, Leiden, The Netherlands; 3 Department of Developmental Psychology, Institute of Psychology, Utrecht University, Utrecht, The Netherlands; 4 Department of Child and Adolescent Psychiatry/Psychology, Erasmus MC, University Medical Centre Rotterdam – Sophia, Rotterdam, The Netherlands; 5 Institute of Education and Child Studies, Leiden University, Leiden, The Netherlands; 6 Faculty of Social and Behavioral Sciences, Leiden University, 2333 AK Leiden, The Netherlands

**Keywords:** Meta-analysis, Observed parent–child interactions, Childhood depression, Adolescent depression

## Abstract

A lack of positive and the presence of negative parenting are frequently mentioned as risk factors for the development and maintenance of childhood and adolescent depression. However, meta-analytic research is mostly based on parenting as perceived by the child or parent, with the potential risk of a reporter bias. Also, specific patterns of observed child behavior among children with childhood depression are often overlooked and there currently is no meta-analysis on this topic. The current meta-analyses integrate the cross-sectional and longitudinal relation between observed parenting and childhood depression, and the cross-sectional relation between observed child behavior and childhood depression. This preregistered study (*k* = 90) includes 350 effect sizes on five observed parenting behaviors and 131 effect sizes on six observed child behaviors in parent–child interactions that were found via PsychINFO, Web of Science, and Proquest Dissertations and Theses (until the end of September 2024). Multilevel meta-analyses show that parental warmth/support and harsh control/criticism co-occur with and precede childhood depression, with small effect sizes, while childhood depression generally did not precede negative parenting. This aligns with previous research based on subjective reports, indicating that low parental warmth and higher criticism may form risk factors for childhood depression. Observed parental autonomy granting, guidance/structure, and parental depressed affect did not relate to childhood depression. Further, children with depression showed more negative affect, reduced autonomy and engagement, illustrating the interactional challenges these families face. Family interventions could validate and address the challenges posed to children as well as parents in families with a child with depression.

**Supplementary Information:**

The online version contains supplementary material available at https://doi.org/10.1007/s10567-026-00572-8.

## Introduction

In the past decades, both in research and society the importance of safe and supportive parenting has been emphasized for child wellbeing. In light of this, a lack of positive parenting and the presence of negative parenting are frequently considered risk factors for the development of childhood depression (defined here as depressive symptoms and disorders until 19 years). However, there are three important gaps in scientific knowledge on the relation between parenting and childhood depression: Existing meta-analytic studies do neither distinguish between observed (i.e., coded by independent raters) and perceived (i.e., subjective reports of child and/or parent) parenting nor between cross-sectional and longitudinal studies, and child behaviors are often overlooked. Subsequently, the conclusions that can be drawn about parenting practices and childhood depression are limited.

The current study addresses these gaps and aims to gain a more detailed understanding of the (directional) relation between observed parent–child interaction behaviors and childhood depression. This is crucial given that depressive disorders are among the most common psychiatric disorders (Alonso et al., 2004; Trimbos, 2023), with prevalence of symptoms first occurring in middle childhood and rising throughout adolescence (Lewinsohn et al., 1998; Ormel et al., 2015; Solmi et al., 2022). Early-onset of depression is associated with serious impairments and long-term risks, including recurrence and suicide attempts (Curry et al., 2011; Fernando et al., 2011; Johnson et al., 2018; Rohde et al., 2013), and high societal costs (Bodden et al., 2022). This highlights the importance to understand underlying processes and ultimately inform more effective prevention and intervention strategies.

### Parenting and Childhood Depression

Research on the relation between parenting and childhood depression has been summarized in several meta-analyses (Goodman et al., 2020; McLeod et al., 2007; Yap & Jorm, 2015; Yap et al., 2014; Zhang & Ji, 2024) and in one review specifically on observed parenting and childhood depression (Chapman et al., 2016). These existing meta-analyses and review provide important insights, but do not quantify the state of research on *observed* parenting as concurrently and prospectively related to childhood depression. The existing meta-analyses, based on a combination of observed and perceived measures, indicate that parental criticism and a lack of warmth are related to childhood depression, showing a small, significant relation, accounting for 3–11% of the variance (McLeod et al., 2007; Yap & Jorm, 2015; Yap et al., 2014). Further, they show that the relation between observed parenting is weaker, although still significant, than the relation of perceived parenting with childhood depression (McLeod et al., 2007; Yap & Jorm, 2015). The (longitudinal) relation between observed parenting and childhood depression has been reviewed by Chapman et al. (2016), indicating the strongest evidence for relations between observed maternal disengagement, parental dysphoria, and low warmth/positivity, across cross-sectional and longitudinal studies. However, these findings have not yet been quantified in a meta-analysis, previous meta-analytic studies do not include dissertations, and are in need for an update.

Combining insights from existing meta-analyses (including observed as well as perceived parenting) (McLeod et al., 2007; Yap & Jorm, 2015; Yap et al., 2014) with insights from the review by Chapman et al. (2016), the strongest evidence has been found for a concurrent and prospective relation between a lack of parental warmth and childhood depression. Furthermore, a consistent relation between parental criticism and childhood depression is evident in meta-analyses that combine observed and perceived parenting, but is less clear in the review on only observed parenting. With the evidence so far, caution is warranted for the conclusion that an *observed* lack of positive and presence of negative parenting *precede* childhood depression and therefore the implications for clinical practice are limited. The current study will quantify and update the state of research on observed parenting as related to childhood depression, distinguish between specific types of parenting, and distinguish between cross-sectional and longitudinal associations.

### Child Behaviors and Childhood Depression

A third gap in meta-analytic research concerns the role of child behavior in parent–child interactions as related to childhood depression. Children are not passive recipients, but actively contribute to interactions with their parents. Moreover, raising children with depression may be challenging for parents (Stapley et al., 2016). Several theories emphasize that behaviors of parents and children mutually influence each other (Bronfenbrenner, 1977; Coyne, 1976). In order to understand the contributions of the child in family dynamics and the challenges posed to families with a child with depression, it is important to also gain insights into the relation between observed child behaviors in parent–child interactions and childhood depression. The review by Chapman et al. (2016) summarizes studies on this topic and shows relatively consistent results for what they referred to as ‘maladaptive emotion regulation’ in children with depression. Results indicated that symptoms of depression are indeed expressed in interactions with their parents, for example by prolonged depressive and aggressive expressions. Further, there were indications of a lack of autonomous behavior among children with depression (Chapman et al., 2016).

### Clinical Status of Childhood Depression as a Moderator

Lastly, it is important to consider the clinical status of the child’s depression as a moderator. The characteristics of a clinical depression include, amongst others, substantial affective symptoms (depressive affect, anhedonia) and impaired social functioning (APA, 2013). A clinical depression may interfere more with parent–child interactions than symptoms of depression. We will test whether the clinical status of the depression moderates the relation of observed parental as well as observed child behaviors with childhood depression and we expect stronger effects in studies examining children with a clinical depression diagnosis than children with depression symptoms. This stronger effect was previously found in the meta-analysis by McLeod et al. (2007) for the relation between parenting and childhood depression, including observed as well as perceived parenting. However, here the stronger effect may be due to negative schemas in the clinical group when reporting on perceived parenting, rather than *observed* parenting, which will be examined in the current study.

## Current Study

In sum, the current study aims to provide a meta-analytic overview on the relation between observed parent–child interaction behaviors and childhood depression. We investigate the role of parenting in studies with both cross-sectional and longitudinal designs, and the role of child behaviors in cross-sectional studies. We hereby aim to provide a more detailed understanding of observable family dynamics and the (longitudinal) relation with childhood depression. More specifically, we expect less positive and more negative observed parenting to relate to more childhood depression, based on cross-sectional studies (H1). We will examine directional relations based on longitudinal studies: We expect less positive and more negative observed parenting to precede more childhood depression (H2a) and we will explore whether more childhood depression precedes less positive and more negative observed parenting (H2b). We further expect less positive and more negative observed child behaviors to relate to more childhood depression, based on cross-sectional studies (H3). We decided not to examine the longitudinal relation for child behaviors, because we conceptualize these child behaviors as proximal manifestations of the depression rather than independent predictors or outcomes. Longitudinal modeling would essentially test whether the depression symptoms or manifestations predict themselves over time, which is not our aim. Next, we expect that the findings are stronger in studies on clinical depressive disorders than depression symptomatology (H4). Lastly, we will explore whether several methodological (e.g., type of interaction task) and sample (e.g., parent and child biological sex) characteristics explain the expected heterogeneity between effect sizes.

## Methods

### Preregistration and Guidelines

This set of meta-analyses was preregistered at OSF (link to preregistration: https://osf.io/38vma) and followed guidelines of the PRISMA statement (Moher et al., 2010).

### Selection of Studies

PsychINFO, Web of Science, and ProQuest Dissertations and Theses were systematically searched for eligible studies on the topic until the end of September 2024. The search was done based on title, abstract, and keywords, using the following terms: moth* or matern* or fath* or patern* or parent* and observ* or lab* or cod* and depress* or dysthym* and adolesc* or youth* or child* or *puberty* or teenage*. Studies were included if (1) they included an assessment of observed behavior of parent and/or child as coded from a dyadic or family interaction task, (2) they included an assessment or diagnosis of childhood depression, (3) the age of the participating children was under 19 years, and (4) the study was written in English, French, German, or Spanish. Studies were excluded if (1) the observed behavior was not between parent and child (e.g., peer and child, or behavior from one parent directed to the other parent in a family interaction task), (2) the measure of observed behavior did not entail a direct measure of behavior of a specific person (e.g., attachment relationship, family environment were excluded), (3) they were intervention studies for which the interaction behavior and childhood depression were not measured at baseline, (4) childhood depression was assessed using the Child Behavior Checklist, Youth Self-Report, or Teacher’s Report Form (e.g., Achenbach, 2018), because the subscales of these questionnaires either combine depressive symptoms with symptoms of anxiety (anxious/depressed) or only focus on part of depressive symptoms (withdrawn/depressed), and (5) if children with depression were compared to a group with another psychiatric disorder, but not to a control group.

In case multiple studies reported on the same sample, we chose the study that (1) reported the most complete overview of behavioral constructs, (2) reported the clearest statistical information, (3) reported on the largest sample, and/or (4) was peer-reviewed. If multiple studies reported on the same sample, but on different aspects (e.g., different behavioral constructs, cross-sectional v. longitudinal), both studies were included.

**Fig. 1:**
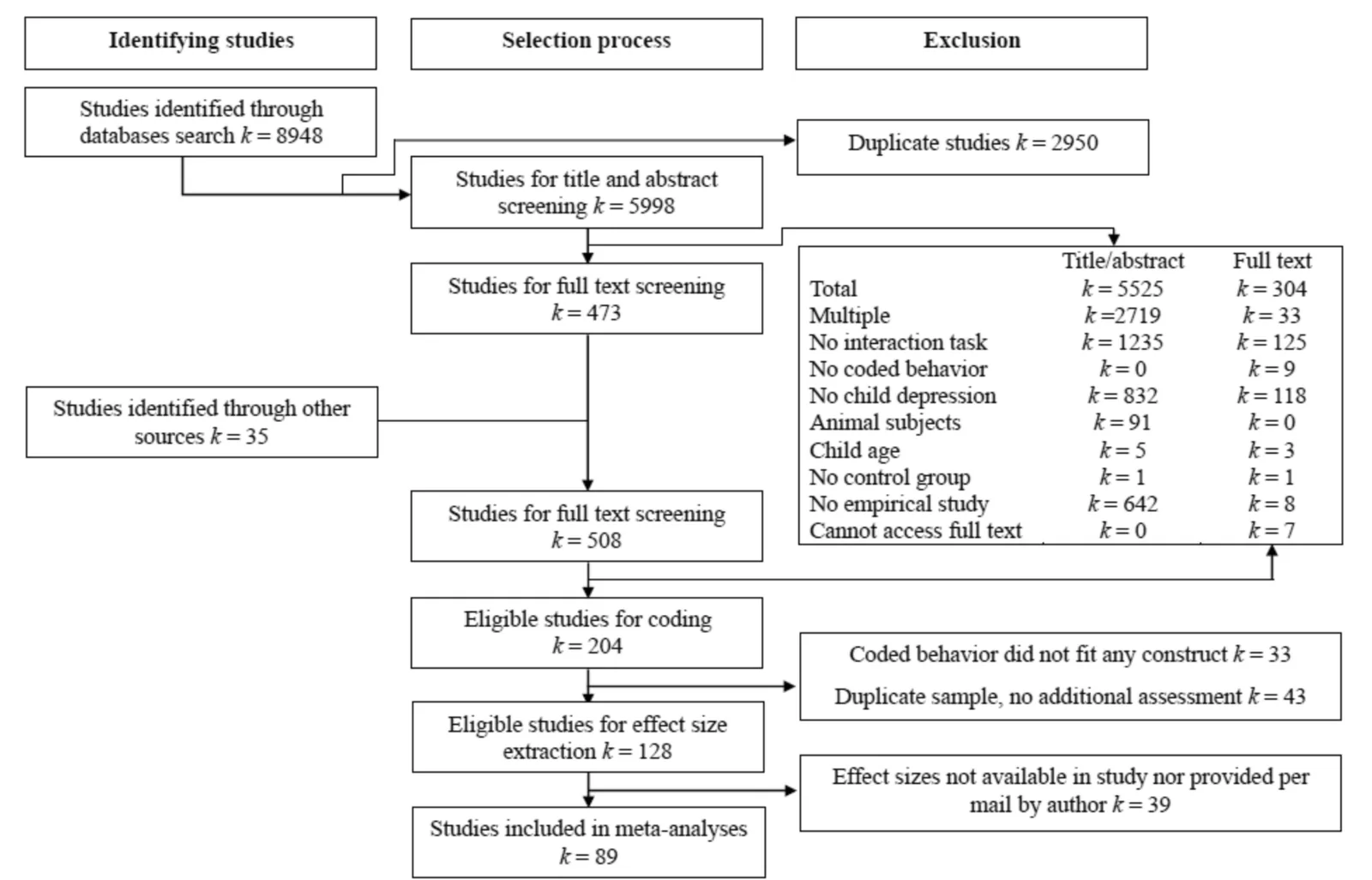
Flowchart selection process

Figure 1 presents the flowchart of the search and selection process. A total of 600 studies were double-coded for screening of titles and abstracts, with good interrater agreement (Cohen’s *κ* = .82). A total of 204 studies were eligible for coding. The final sample in the analyses included 89 studies from 80 unique samples after excluding studies with duplicate samples (unless they examined different assessments or timepoints), with coded behaviors that did not fit any of the constructs (see Coding of the Studies – Behavioral Constructs), and that did not provide relevant effect sizes in the report or by mail.

### Coding of the Studies

The first author and three Clinical Psychology master students coded information on study and sample characteristics (sample size, parents’ and children’s sex, children’s age, ethnicity, interval between assessments of longitudinal studies in years), the assessment of childhood depression (informant, source), the parent–child interaction (setting, task, coding), and the type of comparison (clinical depression v. control, group comparison based on cut-off score of questionnaire, continuous comparison). The coders independently coded a reliability set of thirty studies and showed sufficient to excellent reliability on most of the categorical (Cohen’s *κ* *M* = .79, range [.63, .91]) and continuous (single measures ICC *M* = .98, range [.80, 1.00]) variables. Reliability was insufficient for the variables “ethnicity” (single measures ICC *M* = .68, range [.41, 1.00]) and “type of coding” (micro/sequential/macro) (Cohen’s *κ* *M* = .34, range [.24, .51]). The first author checked the coding of these variables of all studies, and these were only included as descriptives, not in the final analyses.

We developed an assessment of the methodological quality of the studies based on the assessment tool for observational cohort and cross-sectional studies of the National Heart, Lung, and Blood Institute (2021) and the Newcastle–Ottawa Scale (Wells et al., 2021). A total of seven items (see Supplementary Material) were included and scored as lacking in quality (0) or sufficient in quality (1), and summed into one total score, with good to excellent reliability between coders (single measures ICC: *M* = .83).

### Behavioral Constructs

The definitions of the observed behaviors were examined per study and overarching constructs were defined (Table 1). A total of five observed parenting behaviors and six observed child behaviors were defined in discussion between WW, LA, and BE. WW coded all definitions as provided in the studies and discussed unclarities with LA and BE. A reliability set of about 10% of the definitions as provided in the studies were double-coded independently by MM with excellent agreement (Cohen’s κ = .90, *k* = 37).

**Table 1:** Descriptions of constructs

Parental warmth/support	Warmth, positive affect, sensitivity, and supportiveness. Task supportiveness is included here if some warm aspect or child-centeredness was mentioned. We also include ‘promoting relatedness’ of the autonomy-relatedness coding system (Allen et al., 1994), because this is described as “expressing interest or positive reaction toward the other person” (p. 35), and includes validating and engaging behaviors
Parental harsh control/criticism	Active negative behaviors, including harsh control/intrusiveness, criticism, rejection, hostility, and negative affect. Definitions as provided in studies should state something about a negative tone or valence, at least partly directed at the child. We excluded ‘frowns’ (e.g., Dadds et al., 1992), because this can reflect negativity as well as concentration. We also include ‘inhibiting relatedness’ of the autonomy-relatedness coding system (Allen et al., 1994), because this is described as “actively undermining the positive interaction” (p. 43) and includes ignoring/cutting-off and hostile/devaluing behaviors. We excluded psychological control if it was scored on a continuum with autonomy support (Kenney-Benson & Pomerantz, 2005), because the lack of negative behavior does not necessarily indicate the presence of positive behavior
Parental depressed affect	A depressive affective state of the parent, that is not per se directed towards the child. Withdrawal (Buchanan et al., 2022) was not included here, because it concerns specific behavior in relation to child, not parents’ more general depressed affective state
Parental autonomy granting	Granting or promoting autonomous functioning of the child, for example by letting the child make decisions and formulate and further explore their individual input (e.g., thoughts, feelings, decisions)
Parental guidance/structure	Supportiveness in the task, without mentioning child-centeredness and/or positive affect. We excluded ‘skill encouragement’ (Nerenberg, 2014) here, because it is described as “contingent positive reinforcement to teach specific behaviors”
Child positive affect	The child’s affective state being characterized by happiness or friendliness. This can concern the child’s general affective state, or specific expressions of the child towards the parent (e.g., smiling)
Child negative affect	The child’s affective state being characterized by sadness, anxiety, or irritability. This can concern the child’s general affective state, or specific expressions of the child towards the parent (e.g., anger)
Child depressed affect	The child’s affective state being characterized by depressive expressions (e.g., sadness, dysphoria). This can concern the child’s general affective state or specific expressions of the child towards the parent. Solely withdrawal from the interaction is not included here. If depressed affect is combined in coding with other negative affective expressions (e.g., anger), this is included as ‘negative affect’ and not as ‘depressed affect’
Child autonomy	The child’s independent and purposeful functioning, and formulating and exploring of individual input (e.g., thoughts, feelings, decisions)
Child task engagement	The child’s neutral to positive engagement with the task or the interaction with parent
Child unengaged/withdrawal	The child’s behavior of avoidance, seeking distance, withdrawal, or lack of engagement

### Effect Size Extraction

The effect sizes per included construct of the studies were extracted based on as raw as possible data. If possible, bivariate correlations *r* and total sample size were used for continuous comparisons; mean, standard deviation, and sample size per group (depression v. control) were used for group comparisons. The *escalc* function of the *metafor* package in R (Viechtbauer, 2010) and online *psychometrica* calculator (Lenhard & Lenhard, 2022) were used to convert data into *r* (see Supplementary Materials).

Some studies reported (non)significance of effects relevant to the current meta-analysis, but provided no further information (in the reported study, nor via mail). We used a *p*-value of .0009 in case of reported ‘*p* < .001’, a *p*-value of .049 in case of reported ‘*p* < .05’, and a p-value of .50 in case of reported ‘*p* > .05’ or ‘nonsignificant’ (Borenstein et al., 2009). We processed these values in the direction of the effect as reported in the paper, either by explanation in text (e.g., parents of children with depression showed significantly more warmth than controls) or by any reported statistical information. If neither of these options were available in the paper, we processed the *p* value in the same direction as the pooled effect size of the other studies in the meta-analysis.

We contacted several authors to inquire relevant data or effect sizes, in case these were missing in the paper. In case we could not find contact information of the author of a dissertation, we contacted involved supervisors. One author provided additional information for a correct interpretation of the effects (Bureau et al., 2009) (Supplementary Methods). Seven authors provided relevant data or effect sizes (Breaux et al., 2022; Geselowitz et al., 2021; Hayden et al., 2013; Nerenberg, 2014; Tolep, 2014; Vendlinski et al., 2011; Wade et al., 2021). Other authors did not respond to our mails, were unable or unwilling to send the data. The flow with number of studies is presented in Fig. 1 and in text with references in the Supplementary Methods.

### Data Analyses

Meta-analyses were only run if effect sizes of a minimum of three unique samples were available for the given construct. A total of eleven cross-sectional and four longitudinal multilevel meta-analyses were conducted using the *metafor* package (Viechtbauer, 2010) in R (version 4.4.1; R Core Team, 2023). The pooled effects are expressed in Fisher’s *Z* and converted to *r* for interpretation, with .10-.29 as small effects, .30-.49 as medium effects, and of ≥ .50 as large effects (Cohen, 1992). The multilevel models contained three levels: Sampling variance of individual effects (level 1), variance of effect sizes within samples/studies (level 2), and variance between samples/studies (level 3). Analyses were run based on random effects models, because of theoretically assumed heterogeneity between effect sizes. Further, heterogeneity per level is presented in percentages using *I*^*2*^. The significance of variance at the between-samples level (level 3) is tested using a likelihood ratio test comparing the three-level model to a two-level model with between-samples heterogeneity (level 3) constrained to zero. Note that we use the term ‘within/between-samples’ rather than ‘within/between-studies’, because the effect sizes were clustered within unique samples, with some studies reporting on the same sample. The significance of variance at the within-samples level (level 2) is tested using a likelihood ratio test comparing the three-level model to a two-level model with within-samples heterogeneity (level 2) constrained to zero. To check for publication bias of significant relations we used the trim-and-fill procedure based on the two-level model (*rma* argument, *metafor* package), not accounting for clusters of samples. This procedure is not available for three-level meta-analyses, we used the two-level model to get some indication of possible publication bias.

Moderator analyses were performed for the following variables: percentage female parents, percentage female children, age children, quality assessment study, type of task (problem solving, event planning, reminiscence (child shares an emotional experience), teaching task, natural observation (including free play, typical meal)), informant of depression (child, parent, child and parent, other), source of assessment of depression (interview, questionnaire, other), and type of comparison (clinical depression compared to control, group comparison based on cut-off score of questionnaire, continuous comparison). Continuous moderators were only analyzed if effect sizes of minimally three unique samples were available. Categorical moderators were only analyzed if effect sizes of minimally three unique sample for minimally two categories were available. Supplementary Table 1 presents an overview of moderators that were excluded from analyses per construct. The ‘other’ category of the moderators ‘type of task’, ‘informant depression’, and ‘source assessment depression’ was excluded in all moderation analyses, given a small number of effect sizes in most meta-analyses and large variation between studies in the method that was used.

### Deviation from Preregistration

We preregistered to explore whether observed negative conditional parent–child interaction behaviors (e.g., probability of parental criticism in response to child dysphoria) relate to childhood depression by means of a systematic review. Given the large number of constructs of parent and child behaviors, we decided not to include a review on conditional behaviors in the current study, as the extent of this study would become too voluminous.

## Results

The study and participant characteristics are presented in Table 2 and the characteristics of assessing interaction behavior (e.g., type of task) and depression (e.g., informant) in Table 3.

**Table 2:** Study and participant characteristics

					Child				Parent	
Sample	Authors (year)	Country	Design	Interval	N	% female	Mean age (years)	% white	N	% female
1	Allen et al. (2004)	USA	Cross	–	101	51.5	15.9	64.0		100.0
2	Allen et al. (2012)	USA	Cross	–	DEP: 69 HC: 69	66.7				
2	Bodner et al. (2018)	USA	Cross	–	DEP: 43 HC: 50	61.3			DEP: 86 HC: 100	50.0
2	Shortt et al. (2016)	USA	Cross	–	DEP: 47 HC: 60	60.7	16.3	73.5	DEP: 94 HC: 120	50.0
3	Belden et al. (2006)	USA	Cross	–	150	51.3	4.5	87.3		100.0
4	Bender et al. (2007)	USA	Cross	–	131	47.5	15.9	58.0		100.0
5	Brady et al. (2023)	USA	Cross	–	196	39.8	4.7	77.6		
6	Brand-Gothelf et al. (2015)	Israel	Cross	–	DEP: 30 HC: 32	54.8	10.4			
7	Breaux et al. (2022)	USA^a^	Cross	–	DEP: 8 HC: 10	30.0	13.5	38.9	DEP: 8 HC: 10	83.3
8	Bryant (1989)	USA	Cross	–	27	14.8	8.0			100.0
9	Buchanan et al. (2022)	USA^a^	Cross	–	22	69.2	15.3	69.2		80.8
10	Bufferd et al. (2014)	USA	Long	3.0	DEP: 33 HC: 423	46.3	6	86.6	DEP: 33 HC: 423	92.8^a^
11	Bureau et al. (2009)	USA^a^	Long	7.0	45	37.8	8.0	81.0^a^		
			Long	18.0	47	48.9	19.0	81.0^a^		
11	Easterbrooks et al. (2012)	USA	Long	1.0	43	44.2	8.00			100.0
12	Bustos and Santiago (2023)	USA	Cross	–	104	61.0	8.4	0.0		97.0
			Long	0.5	99		8.9	0.0		97.0
			Long	1.0	97		9.4	0.0		97.0
13	Chaplin et al. (2019)	USA	Cross	–	66	48.5	12.6			100.0
14	Cole et al. (1986)	USA	Cross	–	45					
15	Conger et al. (1992)	USA	Cross	–	205	0.0	12.7	100.0		50.0^a^
15	Conger et al. (1993)	USA	Cross	–	220	100.0	12.5	100.0		50.0^a^
15	Reeb and Conger (2011)	USA	Cross	–	298	55.0	17.4	100.0		50.0^a^
16	Cox et al. (2010)	USA	Both	4.0	153	49.7	15			100.0
17	Dadds et al. (1992)	USA	Cross	–	DEP: 18 HC: 16	15.5	10.3			0.0
17	Dadds et al. (1992)	USA	Cross	–	DEP: 18 HC: 16	14.8	10.3			100.0
18	Donohue et al. (2022)	USA	Cross	–	185	33.0	5.2	79.5		100.0
19	Feng et al. (2009)	USA	Cross	–	232	100.0	9.10			100.0
			Long	1.0	225	100.0	10.03			100.0
19	Keenan et al. (2009)	USA	Cross	–	232	100.0	9			100.0
20	Field et al. (1987)	USA^a^	Cross	–	DEP: 20 HC: 18	D: 45.0 C: 44.4	5.2	57.1		100.0
21	Fredrick et al. (2018)	USA	Cross	–	92	62.0	14.2	80.4		100.0
22	Fussner et al. (2015)	USA	Cross	–	134	48.5	13.6			100.0
23	Gaté et al. (2013)	Australia	Cross	–	163		12.5		163	100
			Long	2.6	144	50.0	15.0		144	100
			Long	4.2	120	52.0	16.7		120	100
23	Yap et al. (2010)	Australia	Cross	–	163		12.5	92.0^a^		100.0
23	Yap et al. (2011)	Australia	Cross	–	198	50.0	12.5	92.0	198	100.0
24	Geiss (2016)	USA	Cross	–	31	54.8	13.9	85.7^a^	31	85.7^a^
25	Geselowitz et al. (2021)	USA	Long	11.0	DEP: 20 HC: 110	52.3	T2: 16		DEP: 20 HC: 110	100.0
26	Gjerde et al. (1991)	USA	Long	13.0	88	52.8	18.0		133	54.1
27	Greene et al. (2020)	USA	Long	0.5	242	52.0	5.4	16.0		100.0
28	Griffith et al. (2019)	USA	Long	3.0	498	56.6^a^	14.9^a^	69.1		
28	Griffith et al. (2024)	USA	Long	1.5	595	56.6	13.6	68.3		90.6
29	Gruhn et al. (2016)	USA	Cross	–	93	44.0	12.6	80.6		100.0
			Long	1.0	81	44.0^a^	13.6	80.6^a^		100/0
35	Hamilton et al. (1999)	USA	Cross	–	DEP: 21 HC: 20	D: 24.0 C: 25.0	10.3	85.5		
36	Hayden et al. (2013)	USA	Cross	–	205	53.2	7	87.8	205	100.0
			Long	1.0	181	53.2^a^	8	87.8^a^	181	100.0
			Long	2.0	171	53.2^a^	9	87.8^a^	171	100.0
37	Jacquez (2006) – 3rd graders sample	USA^a^	Cross	–	100		8.62	24.0		
37	Jacquez (2006) – 5th graders sample	USA^a^	Cross	–	100		11.12	24.0		
38	Jacquez et al. (2004)	USA^a^	Both	0.7	72	40.3				100.0
39	Jaser et al. (2008)	USA	Cross	–	72	50.0	12.2			100.0
40	Jodl (1997) – younger sibling	USA	Both	3.0	118		14.5			50.0
40	Jodl (1997) – older sibling	USA	Both	3.0	118		17.0			50.0
41	Katz (1994)	USA	Cross	–	DEP: 19 HC: 82	0.0	12.5		DEP: 28 HC: 119	68.7
42	Katz et al. (2020)	USA	Cross		75	53.1	9.3			100.0
43	Kenney-Benson and Pomerantz (2005)	USA	Cross	–	104	50.0	8.2			100.0
44	Kessler (2012)	USA	Both	4.0	993	50.2	T1: 10.9; T2: 14.9^a^			50.0
45	Kiff et al. (2011)	USA	Cross	–	214	56.0	9.5			100.0
			Long	1.0	189		10.5			100.0
			Long	2.0	189		11.5			100.0
46	Koss et al. (2017)	USA	Cross	–	240	49.2	13.08	73.8		50.0
			Long	1.0	224		14.26			50.0
			Long	2.0	211		15.4			50.0
46	Koss et al. (2018)	USA	Cross	–	206	51.9	13.1	66.5	390	51.5
		USA	Long	1.0	206	51.9	14.2	66.5	390	51.5
		USA	Long	2.0	206	51.9	15.4	66.5	390	51.5
47	Lewandoski & Palermo (2009)	USA	Cross	–	30	63.3	13.5			
48	Little et al. (2015)	Australia	Long	6.0	DEP: 36 HC: 88	52.3	18.7			100.0
48	Schwartz et al., (2012a, 2012b)	Australia	Long	2.6	178	48.9	15.0			100.0^a^
48	Schwartz et al. (2014)	Australia	Long	6.4	113	47.8	18.9			100.0
49	Lougheed et al. (2016)	Canada	Cross	–	72	63.3	16.3			100.0
50	Luby et al. (2006)	USA			DEP: 54 HC: 56	37.6	4.2			100.0
50	Luby et al. (2012)	USA	Cross	–	DEP: 41 HC: 51	52.2				
51	Lynch (2002)	USA	Cross	–	50	48.0	8.6			100.0
52	Lim et al. (2008)	USA	Cross	–	242	46.5	11.2	22.3	242	100.0
52	Lim et al. (2011)	USA	Cross	–	106	36.8	11.4	32.0	106	0
53	Martinez-Torteya (2012)	USA	Long	9.0	119	45.4	10			100.0
54	Messer and Gross (1995)	USA	Cross	–	DEP: 10 HC: 10	60.0	10.1		26	76.9
55	Morris and Oosterhoff (2016)	USA	Cross	–	90	50.0	10.3		180	50.0
56	Nerenberg (2014)	USA	Cross	–	336	53.6	Range too wide		607	51.6
57	Ogbaselase et al. (2022)	USA^a^	Cross	–	92	62.0	14.2	80.4	92	100.0
58	Olino et al. (2016)	USA	Long	1.5	101	57.4	13.0			100.0
59	Oppenheimer et al. (2018)	USA^a^	Cross	–	275	59.0	Range too wide			85.0
			Long	0.5	275	59.0	Range too wide			85.0
			Long	1.5	275	59.0	Range too wide			85.0
60	Parent et al. (2014)	USA	Cross	–	180	49.4	11.5	74.4		88.9
61	Pavlidis et al. (2001)	USA	Cross	–	DEP: 20 HC: 20	50.0	14.3			100.0
62	Pineda et al. (2007)	USA	Cross	–	72	59.7		70.8	72	100.0
63	Richmond et al. (2020)	Australia	Cross	–	155	52.3	8.4	70.3		100.0
64	Sagrestano et al. (2003)	USA	Cross	–	302	56.62	11.0			89.0
			Long	2.0	275	58.5	13.0			89.0
65	Shapiro et al. (2014)	USA	Cross	–	23	52.6^a^	7.79			100.0
66	Sheeber et al. (2007)	USA	Cross	–	DEP: 82 HC: 83	70.3	16.4	86.1	DEP: 123 HC: 130	61.3
67	Sheeber, & Sorenson (1998)	USA	Cross	–	DEP: 26 HC: 26	65.4	15.6		DEP: 26 HC: 26	100.0
68	Siener and Kerns (2012)	USA	Cross	–	87	55.2	11.3	67.0		100.0
69	Slesnick and Waldron (1997)	USA	Cross	–	DEP: 17 HC: 20	78.4	15.1			50.0^a^
70	Smith et al. (2014)	USA	Cross	–	155	51.6	3.8	41.9		93.5^a^
71	Tan et al. (2014)	USA^a^	Cross	–	DEP: 14 HC: 26	62.5	14.8	80.0	DEP: 14 HC: 26	100.0
72	Tolep (2014)	USA	Cross	–	156	50.6	4.2	48.4		93.6
73	Turpyn et al. (2015)	USA	Cross	–	198	49.0	13.3			98.0
74	Valeri (2000)	USA	Cross	–	DEP: 27 HC: 60	38.6				
75	van Doorn et al. (2016)	Canada	Cross	–	111	12.0	9.4	82.0		100
76	Vendlinski et al. (2011)	USA	Cross	–	654	50.2	8.2	94.7		100.0
77	Wade et al. (2021)	Canada	Long	1.5	397	49.3^a^	1.5	56.5^a^	397	100.0
			Long	3.0	385	49.3^a^	3	56.5^a^	385	100.0
78	Workman (2009)	USA	Cross	–	76	50.0	9.7	76.3		85.7
79	Yan et al. (2017)	China	Cross	–	150	42.0	8.54			80.7
80	Zocca (2017)	USA	Cross	–	295					
			Long	1.0	251					

DEP, children with a depressive disorder; HC, healthy control children. We coded the percentage of white participants to provide some insights into the level of Western, educated, industrialized, rich, democratic (WEIRD) participants; the percentage of white participants was the most consistently reported of these characteristics

^a^Best guess based on related information that was reported in the study, or in other studies on the same sample

**Table 3:** Characteristics of assessing observed interaction behavior and childhood depression

		Parent–child interaction measure					Depression measure		
Sample	Authors (year)	Task	Setting	Coding system	Type of coding	Construct label in paper (label in current meta)	Inf	Source	Comp
1	Allen et al. (2004)	PSI	Lab	Autonomy and relatedness (Allen et al., 1998)	Micro aggregated	Child overpersonalizing (aut.), recanting (aut.)	C	Q	Con
2	Allen et al. (2012)	PSI, other	Lab	LIFE	Micro aggregated	Parent proportion of duration, frequency, and duration of angry (crit.) and dysphoric (DEP) behavior	C	I	Gr-cl
2	Bodner et al. (2018)	PSI, other	Lab		Macro	Parent happy affect (warmth); Child angry (NA), dysphoric (DEP), and happy affect (PA)	C	I	Gr-cl
3	Belden et al. (2006)	Teaching task	Lab		Macro	Parent supportive presence (warmth); Child persistence (task), enthusiasm (task), compliance (task)	P	I	Con
4	Bender et al. (2007)	PSI	Lab	Autonomy and relatedness (Allen et al., 1994)	Micro aggregated	Child inhibition and promotion of autonomy (aut.), and inhibition (NA) and promotion (task) of relatedness	C	Q	Con
5	Brady et al. (2023)	Teaching task		Dyadic Parent–Child Interactions in Early Childhood (PCIT-ED)	Micro aggregated	Parent positive affect and behavior (warmth), negative affect and behavior (crit.)	P	I	Gr-cl
6	Brand-Gothelf et al. (2015)	PSI, EPI		Adapted LIFE	Micro aggregated	Child warmth (PA), hostility (NA)	C + P	I	Gr-cl
							C	Q	Con
7	Breaux et al. (2022)	PSI		System of Hersh and Hussong (2009); System for coding affect developed by Rolon-Arroyo et al. (2018)	Macro	Parent supportive (warmth) and unsupportive (crit.) reactions; Child state of negative emotionality (NA)	C + P	I	Gr-cl
8	Bryant (1989)	Natural observation	Home	Standardized Observation Coding System (SOC-III-B)	Sequential	Parent aversiveness (crit.)	C	Q	Con
9	Buchanan et al. (2022)	PSI		Revised version of Iowa Rating Scale	Sequential	Parent support (warmth), conflict (crit.), contempt (cri.); Child conflict (NA), contempt (NA), withdrawal (withd.)	C	Q	Con
10	Bufferd et al. (2014)	Teaching task	Lab		Macro	Parental hostility (crit.)	P	I	Gr-cl
11	Bureau et al. (2009)	Natural observation, other	Home, lab	Coding system by Lyons-Ruth et al. (1997), EAS	Sequential aggregated, macro	Parental involvement (warmth), hostile-intrusive (crit.), sensitivity (warmth)	C	Q	Con
11	Easterbrooks et al. (2012)	Other	Lab	Emotional Availability Scales	Macro	Parental nonhostility (crit.)	C	Q	Con
12	Bustos and Santiago (2023)	Other	Home	Family Interaction Macro-Coding System (FIMS)	Macro	Parental warmth (warmth), supportiveness (warmth)	C	Q	Con
13	Chaplin et al. (2019)	PSI	Lab	Parent-Adolescent Interaction Task (PAIT) coding system	Macro	Parental warmth (warmth), negativity (crit.)	C	Q	Con
14	Cole et al. (1986)	Teaching task	Lab	Combining Lewinsohn (1968), Reid (1978), Forehand et al. (1978), and Gottman et al. (1976) systems	Micro aggregated	Parental positive (warmth) and negative affect (crit.); Child positive (PA) and negative affect (NA)	C + P	I	Gr-cl
15	Conger et al. (1992)	PSI		Iowa Family Interaction Rating Scale^a^	Macro	Parental nurturant/ involved (warmth), hostility (crit.), depressed affect (DEP)	C	Q	Con
15	Conger et al. (1993)	PSI		Iowa Family Interaction Rating Scale^a^	Macro	Parental nurturant/ involved (warmth), hostility (crit.), depressed affect (DEP)	C	Q	Con
15	Reeb and Conger (2011)	Other		Iowa Family Interaction Rating Scale	Macro	Parental warmth (warmth)	C	Q	Con
16	Cox et al. (2010)	Teaching task	Home	Adapted version of system by Gentzler et al. (2005)	Macro	Parental emotion minimization (crit.), encouragement of emotion expression (warmth), problem-focused coping (guid.)	C	Q	Con
17	Dadds et al. (1992)	Typical meal	Home	Depression observation scale	Sequential aggregated	Parental aversive (crit.), positive (warmth), smile (warmth)	C	I, Q	Gr-cl
17	Dadds et al. (1992)	PSI, typical meal	Lab, home	Depression observation scale	Sequential aggregated	Parental positive (warmth), smile (warmth), positive solution (guid.), aversive content (crit.), depressed (DEP) and angry affect (crit.), aversive (crit.)	C	I, Q	Gr-cl
18	Donohue et al. (2022)	Teaching task	Lab	Dyadic Parent–Child Interactions in Early Childhood	Micro aggregated	Parent positive affect and behavior (warmth), negative affect and behavior (crit.)	P	I	Con
19	Feng et al. (2009)	PSI	Lab	Iowa family interaction rating scale	Macro	Child positive (PA) and negative emotion (NA)	C	I	Gr-cl
19	Keenan et al. (2009)	PSI	Lab	Coding system by Melnick & Hinshaw (2000)	Macro	Child disinhibited negative emotion (NA), inhibited emotions (withd.)	C + P	I	Con
20	Field et al. (1987)	Free play			Macro	Parental constructive play (warmth), fantasy play (warmth), smiling/laughing (warmth), contingent responsivity (warmth); Child constructive play (task), fantasy play (task), smiling/laughing (PA), whining/complaining (NA), seeking instruction (task)	Other	Other	Gr-cl
		Teaching task			Macro	Parental remarks of approval (warmth), remarks of disapproval (crit.); Child watching mother’s demonstration (task), asking for help (task)	Other	Other	Gr-cl
21	Fredrick et al. (2018)	EPI	Home or lab	Revised version of system by Gable et al. (2006) and by Kashdan et al. (2013)	Sequential aggregated	Parental active-constructive responses to youth positive affect (warmth)	C	Q	Con
22	Fussner et al. (2015)	PSI, other	Lab	System for coding interactions of family functioning	Macro; Sequential, aggregated	Maternal support (warmth); Child positive affect (PA)	C	Q	Con
23	Gaté et al. (2013)	EPI, PSI	Lab	LIFE	Micro aggregated	Parent positive (warmth) and aggressive (crit.) behavior	C	Q	Con
23	Yap et al. (2010)	EPI, PSI	Lab	LIFE	Micro aggregated	Child positive interpersonal (task)	C	Q	Con
23	Yap et al. (2011)	PSI, EPI	Lab	LIFE	Micro aggregated	Child aggressive (NA), dysphoric (DEP)	C	Q	Con
24	Geiss (2016)	Other	Lab	Modified version of Emotion Socialization Strategies	Sequential aggregated	Parent supportive (warmth), unsupportive (crit.)	C + P	Q	Con
25	Geselowitz et al. (2021)	Other	Lab		Micro aggregated	Parent support (warmth)	C + P	I	Gr-cl
26	Gjerde et al. (1991)	Teaching task	Lab	Parent interaction Q-sort	Macro	Parental positive engagement (warmth), authoritarian control (crit.), cognitive resourcefulness (guid.)	C	Q	Con
27	Greene et al. (2020)	Teaching task	Lab	Parenting Clinical Observation Schedule (P-COS)	Macro	Parental responsive involvement (warmth)		Q	Con
28	Griffith et al. (2019)	PSI	Lab	Based on systems by Melnick and Hinshaw (2000), and NICHD Early Child Care Research Network (1999)	Macro	Parental support (warmth), responsiveness (warmth), conflict (crit.), criticism (crit.)	C + P	I	Gr-cl
28	Griffith et al. (2024)	PSI	Lab	Based on systems by Melnick and Hinshaw (2000), and NICHD Early Child Care Research Network (1999)	Macro	Parental support (warmth), responsiveness (warmth), conflict (crit.), criticism (crit.)	C	Q	Con
29	Gruhn et al. (2016)	REM	Lab	Iowa Family Interaction Rating Scales	Macro	Parental collaborative (warmth), overinvolved/ intrusive (crit.)	C	Q	Con
35	Hamilton et al. (1999)	PSI	Lab	Affective styles interaction coding system (Doane et al., 1981)	Micro aggregated	Parental negative affective style (crit.), harsh criticism (crit.)	C + P	I	Gr-cl
36	Hayden et al. (2013)	Other	Lab	System of Goldsmith (1995)	Macro	Parental criticism (crit.), enjoyment (warmth), sensitivity (warmth), connectedness (warmth), intrusiveness (crit.)	C	Q	Con
37	Jacquez (2006)	Teaching task	Lab	Based on system by Institute for Social and Behavioral Research, Iowa State University	Macro	Parental hostility (crit.), warmth (warmth), encouraging independence (aut-gr.), intrusiveness (crit.)	C + P	Q	Con
38	Jacquez et al. (2004)	PSI	Lab	Newly developed	Sequential aggregated^a^	Parental positive (warmth) and negative (crit.) remarks	P, C	I, Q	Con
39	Jaser et al. (2008)	PSI, REM	Lab	Iowa family interaction rating scale	Macro	Parental sadness (DEP)	C	Q	Con
40	Jodl (1997)	PSI	Home	Autonomy and relatedness (Allen et al., 1994)	Micro aggregated	Parental inhibiting relatedness (crit.); Child inhibiting autonomy (aut.), inhibiting relatedness (NA)	O, C + P	Obs., Q	Con
41	Katz (1994)	PSI	Home or lab	SASB	Micro aggregated	Parental assert and understand (warmth), nurture and protect (warmth), belittle and blame (crit.); Child assert and separate (aut.), defer and submit (aut.), sulk and scurry (NA)	C	Q	Gr-q
42	Katz et al. (2020)	PSI	Lab^a^	Parent and Child Coding System (PACCS; Katz et al., 2007)	Micro aggregated	Parent validation (warmth), dismissing (crit.); Child positivity (PA), negativity (NA)	C	Q	Con
43	Kenney-Benson and Pomerantz (2005)	Teaching task	Lab		Sequential aggregated	Parental negative affect (crit.)	C	Q	Con
44	Kessler (2012) – T1	PSI + EPI	Home (dad), lab (mom)		Macro	Parental warmth and support (warmth), hostility (crit.), respect for autonomy (aut-gr.)	C	Q	Con
44	Kessler (2012) – T2	PSI	Home		Macro	Parental warmth and support (warmth), hostility (crit.), respect for autonomy (aut-gr.)	C	Q	Con
45	Kiff et al. (2011)	PSI, other	Home	Based on systems by Cowan and Cowan (1992), and Lindahl and Malik (2000)	Macro	Parental warmth (warmth), negativity (crit.), guidance and structure (guid.), autonomy granting (aut-gr.)	C	Q	Con
46	Koss et al. (2017)	PSI	Lab	SCIFF	Macro	Child anger and frustration (NA), withdrawal (withd.), opposition/defiance (NA), positive affect (PA)	C	Q	Con
46	Koss et al. (2018)	PSI	Lab	SCIFF	Macro	Parental hostility (crit.)	C	Q	Con
47	Lewandoski & Palermo (2009)	PSI, REM	Lab	Autonomy and intimacy rating system – subscale independence and responsibility taking	Macro	Child autonomy (aut.)	C	Q	Con
48	Little et al. (2015)	PSI, EPI	Lab	LIFE	Micro aggregated	Parental positive (warmth) and aggressive (crit.) behavior	C	I	Gr-cl
48	Schwartz et al., (2012a, 2012b)	EPI, PSI	Lab	LIFE	Micro aggregated	Parental aggressive (crit.), dysphoric (DEP), positive (warmth)	C	Q	Con
48	Schwartz et al. (2014)	EPI, PSI	Lab	LIFE	Micro aggregated	Parental dysphoric behavior (DEP)	C^a^	I	Gr-cl
49	Lougheed et al. (2016)	PSI	Home or school	Specific affect observational coding system; co-regulation observational system	Micro	Parental supportive regulation (warmth), supportive regulation given child emotion (cond.); Child positive (PA) and negative (NA) emotion	C	Q	Con
50	Luby et al. (2006)	Teaching task		Scale by Egeland & Hiester (1995)	Macro	Child enthusiasm (task), negativity (NA), persistence (task), noncompliance (task), affection (PA), avoidance (withd.)	P	I	Gr-cl
50	Luby et al. (2012)	Other	Lab		Micro aggregated	Parental support (warmth)	P	I	Gr-cl
51	Lynch (2002)	Structured play	Lab	DPICS-II	Micro aggregated	Child positive (PA) and negative (NA) behavior, compliance (task), noncompliance (task)	C	Q	Con
52	Lim et al. (2008)	PSI, REM, other	Lab	Iowa Family Interaction Rating Scale	Macro	Parental positive (warmth) and negative (crit.) behaviors	C + P	I	Con
							C	Q	Con
52	Lim et al. (2011)	PSI, REM, other	Lab	Iowa Family Interaction Rating Scale	Macro	Parental negative behaviors (crit.)	C + P	I	Con
							C	Q	Con
53	Martinez-Torteya (2012)	Free play		Ainsworth’s system	Macro	Parental sensitivity (warmth)	P	I	Con
							C	Q	Con
54	Messer and Gross (1995)	Natural observation	Home	A-FICS	Sequential aggregated	Parental positive (warmth), negative (crit.); Child positive (PA), negative (NA)	C + T + peers	Q	Gr-q
55	Morris and Oosterhoff (2016)	Teaching task	Lab	Ádaptation of system by Greco & Morris (2002), Rork & Morris (2009)	Sequential aggregated	Parental physical takeover (crit.), direct command (crit.), verbal instruction (guid.), critical statement (crit.)	C	Q	Con
56	Nerenberg (2014)	PSI	Home	Based on Coder Impressions system (Forgatch et al., 1992)	Macro	Parental harsh discipline (crit.), positive involvement (warmth)	T	Q	Con
57	Ogbaselase et al. (2022)	PSI, EPI	Lab or home	Dyadic Interaction Coding Manual-V2.0	Micro aggregated	Child emotional inertia of negative affect (NA) and of positive affect (PA)	C	Q	Con
58	Olino et al. (2016)	PSI, EPI	Lab	Interactional dimensions coding system revised	Macro	Parental negative behavior (crit.)	C + P	Q	Con
59	Oppenheimer et al. (2018)	Other	Lab	Systems of Corona et al. (2005), and Melby and Conger (1998)	Macro	Parental positive (warmth) and negative (crit.)	C + P	I	Con
							C	Q	Con
60	Parent et al. (2014)	EPI, other	Unknown	Iowa Family Interaction Rating Scales	Macro	Parental positive (warmth) and negative (crit.) composite	C	Q	Con
61	Pavlidis et al. (2001)	PSI	Lab	Autonomy and relatedness (Allen et al., 1994)	Micro aggregated	Parental exhibit relatedness (warmth), inhibit relatedness (crit.); Child exhibit (task) and inhibit (NA) relatedness, exhibit and inhibit autonomy (aut.)	C + P	I	Gr-cl
62	Pineda et al. (2007)	PSI	Lab	New system	Micro	Parental critical (crit.) and positive (warmth) behavior; Child depressive behavior (DEP)	C	Q	Con
63	Richmond et al. (2020)	PSI, EPI	Lab	Family Interaction Macro-coding System (FIMS)	Macro	Parent warmth (warmth), communication (warmth); Child communication/social maturity (aut.), negativity (NA), warmth (PA)	C	Q	Con
64	Sagrestano et al. (2003)	PSI, teaching task	Lab	System by Holmbeck et al. (1995), based on system by Smetana et al. (1991)	Macro	Parent engagement/ collaboration (warmth), positive affect (warmth); Child engagement/ collaboration (task), positive affect (PA)	C	I	Con
65	Shapiro et al. (2014)	REM	Lab^a^	Adapted coding manual of NICHD	Macro	Parent sensitivity and attunement (warmth), warmth and positivity (warmth), engagement (warmth); Child positivity (PA), attentiveness (task), engagement (task)	C	Q	Con
66	Sheeber et al. (2007)	PSI	Lab	LIFE	Micro aggregated	Parental facilitative (warmth), aggressive (crit.)	C	I, Q	Gr-cl
67	Sheeber, & Sorenson (1998)	PSI	Lab	LIFE	Micro aggregated	Parental depressive (DEP), aggressive (crit.), facilitative (warmth), problem solving behaviors (guid.)	C	I	Gr-cl
68	Siener and Kerns (2012)	PSI	Lab		Macro	Child negative affect intensity (NA), involvement (task)	C	Q	Con
69	Slesnick and Waldron (1997)	PSI	Lab	LIFE	Micro aggregated	Child aversive content and affect (NA), and depressive content and affect (DEP)	C	I	Gr-cl
70	Smith et al. (2014)	Teaching task	Lab		Macro	Parental hostility (crit.), support (warmth), intrusiveness (crit.), quality of instruction (guid.)	P	I	Con
71	Tan et al. (2014)	REM	Lab	Adapted Specific Affect coding system (SPAFF)	Micro aggregated	Parent duration of negative affect (crit.)		I	Gr-cl
72	Tolep (2014)	Teaching task	Lab		Macro	Parental hostility (crit.), supportive presence (warmth)	P	I	Con., gr-cl
73	Turpyn et al. (2015)	PSI	Lab	PAIT coding system (Chaplin, 2010)	Macro	Parental criticism (crit.); Child positive (PA) and negative emotion (NA)	C	Q	Con
74	Valeri (2000)	PSI, EPI, teaching task	Lab	Newly developed micro coding system and macro coding system	Micro aggregated, Macro	Parental encouragement (warmth), pleasant (warmth); Child enthusiasm (PA), unengaged (withd.), pleasant (PA)	C + P	I	Gr-cl
75	van Doorn et al. (2016)	PSI	Home	Newly developed system	Macro	Parental warmth (warmth), psychological control (crit.)	C	Q	Con
76	Vendlinski et al. (2011)	Other	Home	Observer impressions of the primary caregiver	Macro	Parental negativity (crit.), positivity (warmth)	P	Q	Con
77	Wade et al. (2021)	Natural observation, teaching task	Home	Parent–child interaction system, coding of attachment related parenting		Parent responsiveness (warmth), negativity (crit.)	P	Q	Con
78	Workman (2009)	Teaching task	Home (5%) or lab (95%)	Toy Box Activity Coding Manual (NICHD-early child care research network; adapted by Egeland & Heister, 1993)	Macro	Parental autonomy granting (aut-gr.), supportive presence (warmth), positive affect (warmth)	C	Q	Con
79	Yan et al. (2017)	PSI, teaching task	Lab		Macro	Parental autonomy support (aut-gr.)	C	Q	Con
80	Zocca (2017)	PSI	Lab	Based on system by Melnick & Hinshaw (2000)	Macro	Parental positive (warmth) and negative (crit.) affect, support (warmth), criticism (crit.), responsiveness (warmth), conflict (crit.)	C	Q	Con

warmth, warmth/support; crit., harsh control/criticism; DA, depressed affect; aut-gr., autonomy granting; guid., guidance/structure; PA, positive affect; NA, negative affect; aut., autonomy; task, task engagement; withd., withdrawal/unengaged; Inf, informant; C, child; P, parent; T, teacher; I, interview; Q, questionnaire; Comp., comparison; Gr-cl., group comparison based on clinical assessment; Gr-q., group comparison based on cut-off score questionnaire; Con., continuous comparison

^a^ best guess

## Observed Parenting

### Warmth/Support

#### Cross-Sectional Studies

The meta-analysis (*k*_*samples*_ = 47, *k*_*ES*_ = 104) indicated that less parental warmth/support relates to more childhood depression, with a small effect size (Fisher’s* z* = − 0.105, *p* = < .001; 95% CI [− 0.140, − 0.070], *r* = − .104). The funnel and forest plots are presented in Supplementary Fig. 1. The likelihood ratio tests showed significant variance between samples (level 3; σ = .007) (χ^2^ = 16.416, *p* < .001, 46.5% total variance explained) and nonsignificant variance within samples (level 2; σ = .001) (χ^2^ = 1.069, *p* = .301, 5.4% total variance explained). The trim-and-fill procedure indicated some publication bias, with four studies missing on the right side of the distribution. The pooled effect (two-level model) including the four filled studies remained significant (Fisher’s *z* = − 0.086, *p* < .001, r = − .086). None of the moderation analyses were significant (Table 4).

**Table 4:** Moderation analyses for the cross-sectional and longitudinal relations between observed parenting and childhood depression

							Regression analysis moderator			Effect size per category		
		*Q* (*df*)	*p*	*k*_*samples*_	*k*_*ES*_		*b*	SE	*p*	Fisher’s *z*	95% CI	*r*
*Parental warmth/support*												
Cross-sectional												
Female parents (per 10%)		0.150 (1)	.699	42	90	Interc	− 0.116	0.033	< .001			
						Pred	0.001	0.003	.699			
Female children (per 10%)		3.009 (1)	.083	44	93	Interc	− 0.062	0.030	.037			
						Pred	− 0.008	0.005	.083			
Age children (per 1 year)		1.499 (1)	.221	39	85	Interc	− 0.052	0.049	.286			
						Pred	− 0.006	0.005	.221			
Quality assessment study		0.200 (1)	.655	47	104	Interc	− 0.140	0.081	.082			
						Pred	0.007	0.016	.655			
Type of task		0.166 (2)	.920	47	104							
Problem solving (intercept)				18	34		− 0.106	0.030	.001	− 0.105	− 0.165, − 0.046	− .105
Event planning				2	3							
Reminiscence				2	4							
Teaching				10	21		0.020	0.048	.687	− 0.086	− 0.160, − 0.012	− .086
Natural observation				4	11		0.003	0.080	.971	− 0.103	− 0.248, 0.043	− .103
Other				15	31							
Informant depression		0.219 (2)	.897	47	104							
Child (intercept)				31	66		− 0.106	0.022	< .001	− 0.106	− 0.149, − 0.064	− .106
Parent				10	17		− 0.011	0.040	.774	− 0.118	− 0.186, − 0.049	− .117
Child and parent				7	10		0.019	0.055	.736	− 0.088	− 0.194, 0.019	− .087
Other				3	11							
Source assessment depression		0.186 (1)	.666	47	104							
Interview (intercept)				18	28		− 0.095	0.030	.002	− 0.095	− 0.154, − 0.035	− .094
Questionnaire				30	67		− 0.015	0.035	.666	− 0.110	− 0.152, − 0.068	− .109
Other				2	9							
Comparison		0.006 (1)	.940	47	104							
Group-clinical (intercept)				13	25		− 0.108	0.045	.017	− 0.108	− 0.197, − 0.019	− .108
Group-questionnaire cut-off				2	6							
Continuous				34	73		0.004	0.048	.940	− 0.104	− 0.142, − 0.067	− .104
Parenting → depression												
Female parents (per 10%)		3.267 (1)	.071	17	56	Interc	0.022	0.075	.770			
						Pred	− 0.015	0.008	.071			
Female children (per 10%)		16.847 (1)	** < .001**	17	58	Interc	0.013	0.040	.751			
						Pred	− 0.023	0.006	< .001			
Age children (per 1 year)		0.704 (1)	.402	16	56	Interc	− 0.166	0.080	.039			
						Pred	0.006	0.007	.402			
Quality assessment study		6.033 (1)	**.014**	18	61	Interc	0.101	0.088	.249			
						Pred	− 0.038	0.015	.014			
Interval assessments (per 1 year)		0.174 (1)	.677	18	61	Interc	− 0.118	0.033	< .001			
						Pred	0.003	0.006	.677			
Type of task		0.584 (1)	.445	18	61							
Problem solving (intercept)				5	16		− 0.102	0.055	.064	− 0.102	− 0.210, 0.006	− .102
Event planning				2	7							
Reminiscence				1	1							
Teaching				3	4		− 0.077	0.100	.445	− 0.179	− 0.343, − 0.014	− .177
Natural observation				2	5							
Other				8	28							
Source assessment depression		3.148 (1)	.076	18	61							
Interview (intercept)				5	8		− 0.030	0.051	.558	− 0.030	− 0.129, 0.069	− .030
Questionnaire				16	53		− 0.090	0.051	.076	− 0.120	− 0.168, − 0.071	− .119
Other				0	0							
Depression → parenting												
Female parents (per 10%)		0.555 (1)	.456	4	7	Interc	− 0.011	0.053	.833			
						Pred	− 0.003	0.004	.456			
Female children (per 10%)		0.883 (1)	.347	4	7	Interc	− 0.371	0.356	.298			
						Pred	0.066	0.070	.347			
Age children (per 1 year)		5.076 (1)	**.024**	3	5	Interc	− 0.147	0.065	.025			
						Pred	0.012	0.005	.024			
Quality assessment study		5.834 (1)	.**016**	4	7	Interc	0.193	0.089	.026			
						Pred	− 0.043	0.018	.016			
Interval assessments (per 1 year)		0.292 (1)	.589	4	7	Interc	− 0.082	0.091	.368			
						Pred	0.025	0.046	.589			
*Parental harsh control/criticism*												
Cross− sectional												
Female parents (per 10%)		7.439 (1)	**.006**	39	88	Interc	0.139	0.025	< .001			
						Pred	− 0.007	0.002	.006			
Female children (per 10%)		0.701 (1)	.402	42	93	Interc	0.068	0.028	.016			
						Pred	0.004	0.005	.402			
Age children (per 1 year)		0.267 (1)	.605	38	90	Interc	0.054	0.053	.313			
						Pred	0.003	0.005	.605			
Quality assessment study		3.981 (1)	**.046**	44	108	Interc	− 0.029	0.060	.636			
						Pred	0.024	0.012	.046			
Type of task		3.121 (2)	.210	43	106							
Problem solving (intercept)				21	40		0.095	0.025	< .001	0.095	0.047, 0.143	.095
Event planning				1	2							
Reminiscence				1	1							
Teaching				10	31		− 0.066	0.038	.079	0.029	− 0.027, 0.085	.029
Natural observation				4	8		− 0.045	0.083	.590	0.050	− 0.105, 0.205	.050
Other				10	26							
Informant depression		2.893 (2)	.235	43	106							
Child (intercept)				28	66		0.107	0.020	< .001	0.107	0.067, 0.146	.106
Parent				8	18		− 0.059	0.037	.109	0.048	− 0.016, 0.112	.048
Child and parent				7	11		0.020	0.056	.722	0.126	0.021, 0.232	.126
Other				5	13							
Source assessment depression		0.232 (1)	.630	43	106							
Interview (intercept)				16	31		0.101	.030	.001	0.101	0.042, 0.161	.101
Questionnaire				28	68		− 0.017	0.035	.630	0.084	0.045, 0.124	.084
Other				3	9							
Comparison		0.906 (1)	.341	43	106							
Group-clinical (intercept)				13	26		0.124	.042	.003	0.124	0.041, 0.207	.124
Group-questionnaire cut-off				2	4							
Continuous				31	78		− 0.043	0.046	.341	0.081	0.045, 0.116	.081
Parenting → depression												
Female parents (per 10%)		6.232 (1)	**.013**	16	49	Interc	− 0.002	0.053	.967			
						Pred	0.015	0.006	.013			
Female children (per 10%)		3.263 (1)	.071	15	47	Interc	0.068	0.039	.078			
						Pred	0.011	0.006	.071			
Age children (per 1 year)		0.038 (1)	.846	15	49	Interc	0.110	0.069	.112			
						Pred	0.001	0.006	.846			
Quality assessment study		2.846 (1)	.092	17	54	Interc	− 0.038	0.095	.689			
						Pred	0.028	0.017	.092			
Interval assessment (per 1 year)		6.191 (1)	**.013**	17	54	Interc	0.166	0.029	< .001			
						Pred	− 0.017	0.007	.013			
Type of task		13.279 (2)	**.001**	17	54							
Problem solving (intercept)				8	24		0.101	0.026	< .001	0.101	0.050, 0.151	.100
Event planning				3	9		0.156	0.045	.001	0.256	0.170, 0.343	.251
Reminiscence				1	1							
Teaching				3	4		− 0.032	0.058	.587	0.069	− 0.033, 0.171	.069
Natural observation				1	2							
Other				5	14							
Informant depression		0.080 (1)	.778	17	54							
Child (intercept)				14	44		0.125	0.024	< .001	0.125	0.077, 0.172	.125
Parent				3	4		0.016	0.055	.778	0.140	0.040, 0.240	.139
Child and parent				1	2							
Other				1	4							
Source assessment depression		0.741 (1)	.389	17	54							
Interview (intercept)				3	5		0.097	0.040	.015	0.097	0.019, 0.175	.097
Questionnaire				15	45		0.032	0.037	.389	0.129	0.085, 0.173	.128
Other				1	4							
Depression → parenting												
Female parents (per 10%)		13.460 (1)	** < .001**	3	5	Interc	0.177	0.069	.011			
						Pred	− 0.016	0.004	< .001			
Female children (per 10%)		18.279 (1)	.618	3	5	Interc	− 0.261	0.602	.665			
						Pred	0.063	0.126	.618			
Quality assessment study		0.255 (1)	.613	3	5	Interc	− 0.066	0.213	.758			
						Pred	0.021	0.041	.613			
Interval assessment (per 1 year)		0.000 (1)	.997	3	5	Interc	0.039	0.124	.754			
						Pred	− 0.0002	0.053	.997			
*Parental autonomy granting*												
Female parents (per 10%)		0.005 (1)	.945	4	7	Interc	− 0.045	0.071	.529			
						Pred	− 0.001	0.007	.945			
Female children (per 10%)		0.323 (1)	.570	4	7	Interc	− 0.406	0.617	.511			
						Pred	0.070	0.123	.570			
Age children (per 1 year)		3.291 (1)	.070	5	13	Interc	0.239	0.173	.166			
						Pred	− 0.027	0.015	.070			
Quality assessment study		1.562 (1)	.211	5	13	Interc	0.081	0.087	.347			
						Pred	− 0.027	0.021	.211			
*Parental guidance/structure*												
Female parents (per 10%)		0.213 (1)	.644	6	7	Interc	− 0.088	0.107	.410			
						Pred	0.005	0.012	.644			
Female children (per 10%)		0.645 (1)	.422	6	7	Interc	0.226	0.335	.501			
						Pred	− 0.051	0.063	.422			
Age children		0.392 (1)	.531	6	7	Interc	0.027	0.115	.818			
						Pred	− 0.007	0.012	.531			
Quality assessment study		0.761 (1)	.383	6	7	Interc	0.182	0.260	.482			
						Pred	− 0.044	0.051	.383			
*Parental depressed affect*												
Female parents (per 10%)		0.740 (1)	.390	5	13	Interc	0.249	0.024	.045			
						Pred	− 0.007	0.008	.390			
Female children (per 10%)		1.605 (1)	.112	5	13	Interc	0.258	0.112	.021			
						Pred	− 0.011	0.009	.205			
Age children		0.084 (1)	.772	4	7	Interc	− 0.016	1.010	.987			
						Pred	0.022	0.077	.772			
Quality assessment study		0.037 (1)	.847	5	13	Interc	0.100	0.558	.858			
						Pred	0.018	0.095	.847			

Significant *p*-values are highlighted in **bold**

*k*_*samples*_, number of unique samples; *k*_*ES*_, number of effect sizes; Interc., intercept; Pred., slope of the predictor variable. Significant moderators are highlighted in bold. The total *k*_*samples*_ and *k*_*ES*_ per categorical moderator include all samples/ES, in order to provide a total overview of prevalence of the categories. Categories within these categorical moderators that were included in analyses are indicated by output in the cells, whereas excluded categories are indicated by empty cells (Fisher’s *z*, 95% CI, *r*). Continuous moderators were only analyzed if effect sizes of minimally three unique samples were available. Categorical moderators were only analyzed if effect sizes of minimally three unique sample for minimally two categories were available. Excluded moderators per construct are presented in Supplementary Table 1

#### Longitudinal Studies: Observed Parenting Preceding Childhood Depression

The meta-analysis (*k*_*samples*_ = 18, *k*_*ES*_ = 61) indicated that less parental warmth/support relates to more subsequent childhood depression, with a small effect size (Fisher’s* z* = -0.108, *p* < .001; 95% CI [− 0.157, − 0.060], *r* = − .108). The funnel and forest plots are presented in Supplementary Fig. 2. The likelihood ratio tests showed nonsignificant variance between samples (level 3; σ = .006) (χ^2^ = 3.377, *p* = .066, 34.0% total variance explained) and significant variance within samples (level 2; σ = .005) (χ^2^ = 9.347, *p* = .002, 29.3% total variance explained). The trim-and-fill procedure indicated some publication bias, with one study missing on the right side of the distribution. The pooled effect (two-level model) including the filled study remained significant (Fish’s *z* = -0.095, *p* < .001,* r* = -.094).

Moderation analyses (Table 4) indicated that the relation between less parental warmth/support and more subsequent childhood depression is stronger in samples with more girls (*b* = − 0.023, *p* < .001). Further, the intercept of study quality indicated that more parental warmth/support related to *more* subsequent childhood depression (intercept: *b* = 0.101, *p* = .249), this relation became weaker as the quality of studies increased (slope: *b* = − 0.038, *p* = .014). No other significant moderators were found.

#### Longitudinal Studies: Childhood Depression Preceding Observed Parenting

The meta-analysis (*k*_*samples*_ = 4, *k*_*ES*_ = 7) indicated no evidence for a relation between childhood depression and subsequent parental warmth/support (Fisher’s* z* = − 0.038, *p* = .409; 95% CI [− 0.144, 0.067], *r* = − .038). The funnel and forest plots are presented in Supplementary Fig. 3. The likelihood ratio tests showed nonsignificant variance between samples (level 3; σ = .004) (χ^2^ = 1.801, *p* = .180, 58.2% total variance explained) and nonsignificant variance within samples (level 2; σ = .000) (χ^2^ = 0.000, *p* = 1.000, 0.0% total variance explained).

Moderation analyses (Table 4) indicated the relation between childhood depression and subsequent less parental warmth/support is stronger for younger children (intercept: *b* = − 0.147, *p* = .025; slope *b* = 0.012, *p* = .024). Further, the intercept of study quality indicated that more childhood depression related to *more* subsequent parental warmth/support for studies with the lowest quality (intercept: *b* = 0.193, *p* = .026), this relation became weaker as the quality of studies increased (slope: *b* = − 0.043, *p* = .016). No other significant moderators were found.

### Harsh Control/Criticism/Hostility

#### Cross-Sectional Studies

The meta-analysis (*k*_*samples*_ = 44, *k*_*ES*_ = 108) indicated that more parental harsh control/criticism relates to more childhood depression, although the effect did not reach the criterion for a small effect size (Fisher’s* z* = 0.089, *p* = .017; 95% CI [0.056, 0.121], *r* = .089). The funnel and forest plots are presented in Supplementary Fig. 4. The likelihood ratio tests showed significant variance between samples (level 3; σ = .008) (χ^2^ = 6.054, *p* = .014, 24.2% total variance explained) and significant variance within samples (level 2; σ = .005) (χ^2^ = 6.056, *p* = .014, 25.7% total variance explained). The trim-and-fill procedure did not indicate publication bias.

Moderation analyses (Table 4) indicated that the relation between parental harsh control/criticism and childhood depression is stronger in samples with more fathers (*b* = − 0.007, *p* = .006), and stronger for higher quality studies (*b* = 0.024, *p* = .046). No other significant moderators were found.

#### Longitudinal Studies: Observed Parenting Preceding Childhood Depression

 The meta-analysis (*k*_*samples*_ = 17, *k*_*ES*_ = 54) indicated that more parental harsh control/criticism relates to more subsequent childhood depression, with a small effect size (Fisher’s* z* = 0.119, *p* = < .001; 95% CI [0.075, 0.162], *r* = .118). The funnel and forest plots are presented in Supplementary Fig. 5. The likelihood ratio tests showed significant variance between samples (level 3; σ = .005) (χ^2^ = 10.389, *p* = .001, 49.6% total variance explained) and nonsignificant variance within samples (level 2; σ = .000) (χ^2^ = 0.000, *p* = 1.000, 0.0% total variance explained). The trim-and-fill procedure did not indicate publication bias.

Moderation analyses (Table 4) indicated a stronger relation between parental harsh control/criticism and subsequent childhood depression in samples with more mothers (*b* = 0.015, *p* = .013), in studies with a shorter interval between assessments (*b* = − 0.019, *p* = .005), and in the event planning task (v. problem solving task, reference category) (*b* = 0.156, *p* = .001). No other significant moderators were found.

#### Longitudinal Studies: Childhood Depression Preceding Observed Parenting

 The meta-analysis (*k*_*samples*_ = 3, *k*_*ES*_ = 5) showed no evidence for a relation between childhood depression and subsequent parental harsh control/criticism (Fisher’s* z* = 0.040, *p* = .448; 95% CI [-0.091, 0.170], *r* = .040). The funnel and forest plots are presented in Supplementary Fig. 6. The likelihood ratio tests showed nonsignificant variance between samples (level 3; σ = .000) (χ^2^ = 0.000, *p* = 1.000, 0.0% total variance explained) and significant variance within samples (level 2; σ = .007) (χ^2^ = 10.225, *p* = .001, 74.1% total variance explained).

Moderation analyses (Table 4) indicated a stronger, positive relation between childhood depression and subsequent parental harsh control/criticism in samples with more fathers (*b* = − 0.016, *p* < .001). No other significant moderators were found.

### Autonomy Granting

#### Cross-Sectional Studies

 The multilevel meta-analysis (*k*_*samples*_ = 5, *k*_*ES*_ = 13) showed no evidence for a relation between parental autonomy granting and childhood depression (Fisher’s* z* = − 0.017, *p* = .601; 95% CI [−0.086, 0.052],* r* = − .017). The funnel and forest plots are presented in Supplementary Fig. 7. The likelihood ratio tests showed nonsignificant variance between samples (level 3; σ = .000) (χ^2^ = 0.000, *p* = 1.000, 0.0% total variance explained) and significant variance within samples (level 2; σ = .008) (χ^2^ = 8.995, *p* = .003, 72.4% total variance explained). None of the moderation analyses were significant (Table 4).

#### Longitudinal Studies

 Given an insufficient number of samples, we were unable to run meta-analyses on the longitudinal relation from observed autonomy granting to childhood depression (*k*_*samples*_ = 2, *k*_*ES*_ = 4) and from childhood depression to observed autonomy granting (*k*_*samples*_ = 1, *k*_*ES*_ = 2).

### Guidance/Structure

#### Cross-Sectional Studies

 The meta-analysis (*k*_*samples*_ = 6, *k*_*ES*_ = 7) showed no evidence for a relation between parental guidance/structure and childhood depression (Fisher’s* z* = − 0.042, *p* = .299; 95% CI [− 0.132, 0.048], *r* = − .042). The funnel and forest plots are presented in Supplementary Fig. 8. The likelihood ratio tests showed nonsignificant variance between samples (level 3; σ = 0.000) (χ^2^ = 0.000, *p* = 1.000, 0.0% total variance explained) and nonsignificant variance within samples (level 2; σ = 0.00) (χ^2^ = 0.000, *p* = 1.000, 0.0% total variance explained). None of the moderation analyses were significant (Table 4).

#### Longitudinal Studies

 No studies examined the longitudinal relation between observed guidance/structure and adolescent depression.

### Depressed Affect

#### Cross-Sectional Studies

 The meta-analysis (*k*_*samples*_ = 5, *k*_*ES*_ = 13) showed no clear evidence for a relation between parental depressed affect and childhood depression (Fisher’s* z* = 0.196, *p* = .080; 95% CI [-0.027, 0.419], *r* = .193). The funnel and forest plots are presented in Supplementary Fig. 9. The likelihood ratio tests showed nonsignificant variance between samples (level 3; σ = .034) (χ^2^ = 3.224, *p* = .073, 68.2% total variance explained) and nonsignificant variance within samples (level 2; σ = .004) (χ^2^ = 1.002, *p* = .317, 8.0% total variance explained). None of the moderation analyses were significant (Table 4).

#### Longitudinal Studies

 Given an insufficient number of samples, we were unable to run meta-analyses on the longitudinal relation from observed depressed affect to childhood depression (*k*_*samples*_ = 1, *k*_*ES*_ = 6) and from childhood depression to observed depressed affect (*k*_*samples*_ = 0, *k*_*ES*_ = 0).

## Observed Child Behaviors

### Positive Affect

The meta-analysis (*k*_*samples*_ = 19, *k*_*ES*_ = 27) showed a significant negative relation (Fisher’s* z* = − 0.052, *p* = .026; 95% CI [− 0.097, − 0.007], *r* = − .052), indicating that less child positive affect related to more childhood depression at the same timepoint, but did not reach the criterion for a small effect size (inconclusive result H3). The funnel and forest plots are presented in Supplementary Fig. 10. The likelihood ratio tests showed nonsignificant variance between samples (level 3; σ = 0.000) (χ^2^ = 0.000, *p* = 1.000, 0.0% total variance explained) and nonsignificant variance within samples (level 2; σ = 0.000) (χ^2^ = 0.000, *p* = 1.000, 0.0% total variance explained). The trim-and-fill procedure did not indicate publication bias. None of the moderation analyses were significant (Table 5).

**Table 5:** Moderation analyses for the cross-sectional relations between observed child behavior and childhood depression

							Regression analysis moderator			Effect size per category		
		*Q* (*df*)	*p*	*k*_*samples*_	*k*_*ES*_		*b*	SE	*p*	Fisher’s *z*	95% CI	*r*
*Child positive affect*												
Female parents (per 10%)		0.026 (1)	.873	17	20	Interc	− 0.069	0.167	.678			
						Pred	0.003	0.018	.873			
Female children (per 10%)		0.146 (1)	.703	18	26	Interc	− 0.022	0.082	.787			
						Pred	− 0.005	0.014	.703			
Age children		2.047 (1)	.153	17	23	Interc	− 0.207	0.111	.063			
						Pred	0.013	0.009	.153			
Quality assessment study		2.294 (1)	.130	19	27	Interc	0.168	0.147	.253			
						Pred	− 0.039	0.026	.130			
Type of task		0.347 (1)	.556	19	27							
Problem solving (intercept)				9	10		− 0.048	0.037	.198	− 0.048	− 0.122, 0.025	− .048
Event planning				2	3							
Reminiscence				1	1							
Teaching				2	2							
Natural observation				4	5		− 0.071	0.120	.556	− 0.119	− 0.342, 0.105	− .118
Other				4	6							
Informant depression		0.0003 (1)	.987	19	27							
Child (intercept)				14	18		− 0.048	0.023	.032	− 0.048	− 0.093, − 0.004	− .048
Parent				2	2							
Child and parent				3	5		0.002	0.131	.987	− 0.046	− 0.300, 0.208	− .046
Other				2	2							
Source assessment depression		1.096 (1)	.295	19	27							
Interview (intercept)				6	9		− 0.102	0.053	.057	− 0.102	− 0.206, 0.003	− .101
Questionnaire				12	15		0.061	0.059	.295	− 0.040	− 0.088, 0.007	− .040
Other				2	3							
Comparison		0.140 (1)	.709	19	27							
Group-clinical (intercept)				7	11		− 0.079	0.082	.335	− 0.079	− 0.241, 0.082	− .079
Group-questionnaire cut-off				1	1							
Continuous				12	15		0.032	0.085	.709	− 0.047	− 0.092, − 0.003	− .047
*Child negative affect*												
Female parents (per 10%)		0.056 (1)	.813	23	40	Interc	0.134	0.111	.226			
						Pred	− 0.003	0.012	.813			
Female children (per 10%)		0.486 (1)	.486	24	41	Interc	0.204	0.100	.041			
						Pred	− 0.012	0.017	.486			
Age children		0.176 (1)	.675	25	45	Interc	0.187	0.136	.171			
						Pred	− 0.005	0.011	.675			
Quality assessment study		0.812 (1)	.368	26	46	Interc	0.272	0.157	.082			
						Pred	− 0.026	0.029	.368			
Type of task		2.851 (2)	.240	26	46							
Problem solving (intercept)				20	32		0.146	0.035	< .001	0.146	0.078, 0.214	.145
Event planning				4	7		− 0.077	0.046	.092	0.069	− 0.032, 0.170	.069
Reminiscence				0	0							
Teaching				2	2							
Natural observation				4	4		− 0.019	0.132	.889	0.128	− 0.123, 0.378	.127
Other				1	1							
Informant depression		0.219 (1)	.640	26	46							
Child (intercept)				18	32		0.145	0.035	< .001	0.145	0.077, 0.211	.144
Parent				1	1							
Child and parent				6	7		0.033	0.071	.640	0.178	0.037, 0.318	.176
Other				3	6							
Source assessment depression		0.312 (1)	.576	26	46							
Interview (intercept)				10	13		0.115	0.065	.077	0.115	− 0.013, 0.242	.114
Questionnaire				14	25		0.041	0.074	.576	0.156	0.082, 0.231	.155
Other				3	8							
Comparison		0.001 (1)	.977	26	46							
Group-clinical (intercept)				11	15		0.135	.073	.062	0.135	− 0.007, 0.277	.134
Group-questionnaire cut− off				2	3							
Continuous				14	28		0.002	.079	.977	0.137	0.069, 0.206	.137
*Child depressed affect*												
Female parents (per 10%)		0.290 (1)	.591	6	12	Interc	0.563	0.621	.364			
						Pred	− 0.035	0.065	.591			
Female children (per 10%)		0.092 (1)	.761	5	11	Interc	0.137	0.450	.761			
						Pred	0.023	0.076	.761			
Age children		0.595 (1)	.441	4	10	Interc	− 0.533	1.077	.621			
						Pred	0.060	0.078	.441			
Quality assessment study		0.073 (1)	.788	6	12	Interc	0.430	0.718	.549			
						Pred	− 0.037	0.136	.788			
*Child autonomy*												
Female parents (per 10%)		0.149 (1)	.700	6	15	Interc	− 0.170	0.178	.339			
						Pred	0.008	0.020	.700			
Female children (per 10%)		1.437 (1)	.231	6	12	Interc	0.076	0.193	.696			
						Pred	− 0.048	0.040	.231			
Age children		7.608 (1)	**.006**	7	16	Interc	− 0.560	0.166	.001			
						Pred	0.033	0.012	.006			
Quality assessment study		0.762 (1)	.383	7	16	Interc	− 0.310	0.213	.145			
						Pred	0.034	0.039	.383			
*Child task engagement*												
Female parents (per 10%)		1.473 (1)	.225	12	24	Interc	0.624	0.604	.301			
						Pred	− 0.075	0.062	.225			
Female children (per 10%)		0.424 (1)	.515	11	23	Interc	0.007	0.192	.972			
						Pred	− 0.024	0.037	.515			
Age children		0.808 (1)	.369	12	24	Interc	− 0.159	0.006	.010			
						Pred	0.005	0.006	.369			
Quality assessment study		0.119 (1)	.730	12	24	Interc	− 0.176	0.205	.392			
						Pred	0.012	0.036	.730			
Type of task		0.123 (1)	.726	12	24							
Problem solving (intercept)				6	7		− 0.099	0.048	.038	− 0.099	− 0.193, − 0.005	− .099
Event planning				0	0							
Reminiscence				1	2							
Teaching				3	8		− 0.026	0.074	.726	− 0.125	− 0.237, − 0.014	− .125
Natural observation				2	6							
Other				1	1							
Source assessment depression		4.457 (1)	**.035**	12	24							
Interview (intercept)				4	9		− 0.172	0.040	< .001	− 0.172	− 0.250, − 0.094	− .170
Questionnaire				6	8		0.113	0.054	.035	− 0.059	− 0.129, 0.012	− .059
Other				2	7							
Comparison		0.657 (1)	.418	12	24							
Group-clinical (intercept)				5	13		− 0.153	0.065	.019	− 0.153	− 0.028, − 0.025	− .152
Group-questionnaire cut-off				0	0							
Continuous				7	11		0.059	0.073	.418	− 0.094	− 0.157, − 0.032	− .094
*Child withdrawal/unengaged*												
Female parents (per 10%)		0.788 (1)	.375	4	5	Interc	− 0.071	0.155	.647			
						Pred	0.016	0.018	.375			
Female children (per 10%)		0.888 (1)	.346	5	6	Interc	− 0.056	0.129	.664			
						Pred	0.015	0.015	.346			
Age children (per 1 year)		0.688 (1)	.407	4	5	Interc	0.200	0.171	.243			
						Pred	− 0.013	0.016	.407			
Quality assessment study		0.138 (1)	.711	5	6	Interc	− 0.076	0.368	.837			
						Pred	0.023	0.063	.711			

Significant *p*-values are highlighted in **bold**

*k*_*samples*_, number of unique samples; *k*_*ES*_, number of effect sizes; Interc., intercept; Pred., slope of the predictor variable. Significant moderators are highlighted in bold. The total *k*_*samples*_ and *k*_*ES*_ per categorical moderator include all samples/ES, in order to provide a total overview of prevalence of the ategories. Categories within these categorical moderators that were included in analyses are indicated by output in the cells, whereas excluded categories are indicated by empty cells (Fisher’s *z*, 95% CI, *r*). Continuous moderators were only analyzed if effect sizes of minimally three unique samples were available. Categorical moderators were only analyzed if effect sizes of minimally three unique sample for minimally two categories were available. Excluded moderators per construct are presented in Supplementary Table 1

### Negative Affect

The meta-analysis (*k*_*samples*_ = 26, *k*_*ES*_ = 46) indicated that more child negative affect related to more childhood depression, with a small effect size (Fisher’s* z* = 0.134, *p* < .001; 95% CI [0.072, 0.195], *r* = .130). The funnel and forest plots are presented in Supplementary Fig. 11. The likelihood ratio tests showed significant variance between samples (level 3; σ = .010) (χ^2^ = 4.185, *p* = .041, 43.1% total variance explained) and nonsignificant variance within samples (level 2; σ = 0.000) (χ^2^ = 0.000, *p* = 1.000, 0.0% total variance explained). The trim-and-fill procedure did not indicate publication bias. None of the moderation analyses were significant (Table 5).

### Depressed Affect

The meta-analysis (*k*_*samples*_ = 6, *k*_*ES*_ = 12) indicated that more observed child depressed affect related to more childhood depression, with a small effect size (Fisher’s* z* = 0.232, *p* = .037; 95% CI [0.016, 0.447], *r* = .228). The funnel and forest plots are presented in Supplementary Fig. 12. The likelihood ratio tests showed significant variance between samples (level 3; σ = .031) (χ^2^ = 5.769, *p* = .016, 75.1% total variance explained) and nonsignificant variance within samples (level 2; σ = .000) (χ^2^ = 0.000, *p* = 1.000, 0.0% total variance explained). The trim-and-fill procedure indicated publication bias, with five studies missing on the left side of the distribution. The pooled effect including the five filled studies dropped to non-significance (Fisher’s *z* = 0.027, *p* = .660, *r* = .026). It should be noted that the trim-and-fill procedure is based on the two-level model, and the initial effect of the two-level model (Fisher’s *z* = 0.104, *p* = .034, *r* = .103) was considerably smaller than the initial effect of the three-level model. The conclusion on the estimated effect of publication bias is therefore inconclusive. None of the moderation analyses were significant (Table 5).

### Autonomy

The meta-analysis (*k*_*samples*_ = 7, *k*_*ES*_ = 16) indicated that less child autonomy relates to more childhood depression, with a small effect size (Fisher’s* z* = − 0.132, *p* = .029; 95% CI [− 0.249, − 0.016]). The funnel and forest plots are presented in Supplementary Fig. 13. The likelihood ratio tests showed nonsignificant variance between samples (level 3; σ = .010) (χ^2^ = 1.310, *p* = .252, 35.3% total variance explained) and nonsignificant variance within samples (level 2; σ = .000) (χ^2^ = 0.000, *p* = 1.000, 0.0% total variance explained). The trim-and-fill procedure did not indicate publication bias.

Moderation analyses (Table 5) indicated that the negative relation between child autonomy and childhood depression is stronger in samples with younger children (*b* = 0.033, *p* = .006). No other significant moderators were found.

### Task Engagement

The meta-analysis (*k*_*samples*_ = 12, *k*_*ES*_ = 24) indicated that less task engagement related to more childhood depression, with a small effect size (Fisher’s* z* = -0.106, *p* = .001; 95% CI [− 0.164, − 0.048]). The funnel and forest plots are presented in Supplementary Fig. 14. The likelihood ratio tests showed nonsignificant variance between samples (level 3; σ = .001) (χ^2^ = 0.105, *p* = .746, 4.1% total variance explained) and nonsignificant variance within samples (level 2; σ = .000) (χ^2^ = 0.000, *p* = 1.000, 0.0% total variance explained). The trim-and-fill procedure did not indicate publication bias.

Moderation analyses (Table 5) indicated that the relation between child task engagement and childhood depression is stronger in studies assessing depression with interviews (reference category) (*r* = − .170) than questionnaires (*b* = 0.113, *p* = .035; *r* = − .059). No other significant moderators were found.

### Withdrawal/Unengaged

The meta-analysis (*k*_*samples*_ = 5, *k*_*ES*_ = 6) showed no evidence for a relation between child withdrawal/unengaged behavior and childhood depression (Fisher’s* z* = 0.060, *p* = .185; 95% CI [− 0.040, 0.160], *r* = .060). The funnel and forest plots are presented in Supplementary Fig. 15. The likelihood ratio tests showed nonsignificant variance between samples (level 3; σ = .000) (χ^2^ = 0.000, *p* = 1.000, 0.0% total variance explained) and nonsignificant variance within samples (level 2; σ = .001) (χ^2^ = 0.028, *p* = .868, 6.6% total variance explained). None of the moderation analyses were significant (Table 5).

## Discussion

The current study aimed to provide a meta-analytic overview on the relation between observed parental and child interaction behaviors and childhood depression. The key findings can be summarized as follows (see Table 6 for overview): Observed parental lack of warmth/support and harsh control/criticism relate to concurrent and subsequent childhood depression, whereas this was not the case for parental autonomy granting, parental guidance/structure, and parental depressed affect. This suggests that parental lack of warmth/support and the presence of harsh control/criticism may form risk factors for childhood depression. Interestingly, childhood depression generally did not predict subsequent observed parenting, except within specific subgroups (families with younger children for warmth, fathers for criticism). Further, children with depression displayed more negative behaviors, with sound evidence for child negative affect, lower autonomy, and lower task engagement. Finally, we found no evidence for differences between studies examining clinical depression as compared to depression symptomatology.

**Table 6:** Summary of results per construct

Construct		*k*_*samples*_	*k*_*ES*_	*r*	Trim-and-fill	Interpretation significant moderators
Observed parenting						
*Warmth/support*						
Cross-sectional		47	104	− .104***	Four studies filled on right side After filling: *r* = − .086***	No significant moderators
Parenting → depression		18	61	− .108***	One study filled on right side After filling: *r* = − .094***	Stronger effect for female children Stronger effect in positive direction for lower quality studies, stronger effect in negative direction for higher quality studies
Depression → parenting		4	7	− .038	Not applicable	Stronger effect for younger children Stronger effect in positive direction for lower quality studies, stronger effect in negative direction for higher quality studies
*Harsh control/criticism*						
Cross-sectional		44	108	.089*	No studies filled	Stronger effect for male parents Stronger effect for higher quality studies
Parenting → depression		17	54	.118***	No studies filled	Stronger effect for female parents Stronger effect for shorter interval Stronger effect in event planning task
Depression → parenting		3	5	.040	Not applicable	Stronger effect for male parents
Autonomy granting		5	13	− .017	Not applicable	No significant moderators
Guidance/structure		6	7	− .042	Not applicable	No significant moderators
Depressed affect		5	13	.193	Not applicable	No significant moderators
Observed child behaviors						
Positive affect		19	27	− .052*	No studies filled	No significant moderators
Negative affect		26	46	.130***	No studies filled	No significant moderators
Depressed affect		6	12	.228*	Five studies filled on left side After filling: *r* = .026^a^	No significant moderators
Autonomy		7	16	− .132*	No studies filled	Stronger effect for younger children
Task engagement		12	24	− .106**	No studies filled	Stronger effect interview assessment depression
Withdrawal/unengaged		5	6	.060	Not applicable	No significant moderators

Effect sizes that were significant *and* reached the criterion for a small effect (r ≥ .10) are presented in **bold** * *p* < .05; ** *p* < .01; *** *p* < .001

^a^ The trim-and-fill procedure is done based on the two-level model. For the relation between observed child depressive affect and childhood depression, the initial effect of the two-level model (*r* = .103*) was considerably smaller than the initial effect of the three-level model (*r* = .228*)

### Observed Parenting

It can be concluded that childhood depression is more prevalent among families with parents who display relatively low observed parental warmth and support and more harsh control and criticism based on a large set of effect sizes (ranging between 54 and 106 per analysis), also when these parenting behaviors are observed prior to the development of the depression (or depressive symptoms). Previous meta-analytic research clearly showed the link between perceived parental lack of warmth and presence of criticism with childhood depression (McLeod et al., 2007; Yap & Jorm, 2015; Yap et al., 2014), which is herewith extended to observed parental warmth and criticism. The current effect sizes are small and heterogeneous. The heterogeneity indicates that the severity of lack of warmth and criticism may greatly differ among families. The small effect sizes mean that parenting does not fully explain the onset and maintenance of childhood depression and suggest that other factors also play a role, which has been previously indicated by epidemiological (Dobson & Dozois, 2011) and qualitative (e.g., Wentholt et al., 2024) research. Still, the patterns of significant concurrent and prospective relations that emerged in this meta-analysis seem to indicate that actual parenting behaviors (warmth/support, harsh control/criticism), and not only the perception of these behaviors by children or parents, relate to the development of childhood depression.

The importance of parental warmth and support for the mental well-being of children has been underscored by key scholars and many therapists in the field. It has been linked to fostering a secure attachment (Bowlby, 1979), building self-esteem and social competence in children (Kim & Cicchetti, 2004), and functions as a buffer against stress and negative life events (Hostinar et al., 2014). These concepts are all relevant in the context of depression and a chronic lack of parental warmth may have lifetime pervasive effects.

The longitudinal relation between a lack of observed parental warmth/support and increased childhood depression was stronger among girls. This is in line with previous research showing that girls may internalize a lack of warmth more strongly (Hankin & Abramson, 2001), but also that the presence of warmth and positive self-perceptions in the interpersonal domain decreases the likelihood of depression among girls (Eberhart et al., 2006). Gender socialization may play an important role here, with research for instance showing more emotional burdening and more emphasis on responsibility in socializing girls (Hankin & Abramson, 2001).

Even though there was no main effect in the relation from childhood depression to subsequent observed parental warmth/support, this was moderated by child age (range included studies: 3–14 years) and appears to be relevant particularly for families with young children. Previous studies described lowered parental warmth and support in response to their child’s depression as support erosion (e.g., Branje et al., 2010); as also described in the social interaction theory (Coyne, 1976). The current meta-analysis suggests that this process of warmth and support erosion is specifically relevant among families with young children, where caretaking demands may be higher and parenting skills and confidence potentially less developed compared to parents with older children.

In addition to the role of parental warmth/support, the current set of meta-analyses provided insights into the relation between parental harsh control/criticism and childhood depression. Parental expressions of harsh control/criticism towards the child’s characteristics, thoughts, and feelings can undermine the child’s self-worth, dysregulate thoughts and feelings, exacerbate emotional problems, and diminish the ability to establish emotional links with others (Barber, 1996; Bolton et al., 2009; Goagoses et al., 2023; Ryan & Deci, 2000; Sloover et al., 2023; Soenens et al., 2019). Previous meta-analyses, that mainly included perceived (and a few studies on observed) criticism, showed a clear relation with childhood depression (McLeod et al., 2007; Yap & Jorm, 2015; Yap et al., 2014), whereas results in the review on observed criticism were inconclusive (Chapman et al., 2016). The current meta-analyses shed light on these inconsistencies. We found a significant relation between parental harsh control and criticism and depression when assessed at the same time, even though the association was negligible in size. We further found that observed criticism preceded subsequent childhood depression (with a small effect size) and significant moderators for both relations. This indicates that observed parental criticism is more prevalent among families with children with depression, that the prospective relation is slightly stronger, and that there is variation among families (i.e., biological sex parent) and contexts (i.e., type of task), as outlined below.

Interestingly, the cross-sectional and child-directed longitudinal (from depression to criticism) relations were stronger among fathers, whereas the parent-directed longitudinal (from criticism to depression) relation was stronger among mothers. These findings suggest that particularly fathers may respond to their child’s depression in a criticizing manner whereas criticism by mothers may have a stronger effect on children in terms of development of depressive symptoms. The increased frequency of criticism by fathers does not seem to precede the depression, but is mostly observed during actual increased symptoms of depression of the child, as indicated by the cross-sectional effect and, with caution (one study reported separate effects per sex, others only included mothers), even over time.

Further, the relation from observed criticism to subsequent childhood depression was moderated by the type of interaction task, with stronger effects in the event planning task than the problem solving task. Thus, parental criticism in a positive context appears to be more predictive of subsequent childhood depression. Parents tend to show less positive and more negative behavior in more demanding, challenging interactions (e.g., problem solving) than in more collaborative, positive interactions (e.g., event planning) (e.g., Branger et al., 2019; Wentholt et al., 2025). Parental criticism expressed in interactions that provide opportunities for collaboration and positivity may therefore have a stronger predictive value for the development of depressive symptoms.

We did not find evidence for relations of observed parental autonomy granting, guidance/structure, and depressed affect with childhood depression. The lack of a significant relation of parental autonomy granting is notable, given that Yap et al. (2014) found small to medium cross-sectional and longitudinal effects in their meta-analysis on adolescent depression. The current meta-analysis shows that this does not replicate to *observed* autonomy granting. It seems that adolescents who perceive their parents as being less autonomy granting, experience (longitudinally) more depression, but parents of these adolescents do not show less autonomy granting behaviors. It is possible that the effects of a lack of autonomy granting (e.g., erosion of child self-esteem and -reliance) do not occur directly, but rather over time. This could not be tested in the current meta-analysis given an insufficient number of effect sizes and provides an avenue for future research.

Three final aspects should be mentioned with regards to observed parenting. Parenting has traditionally been mainly researched in mothers. Some of the studies in the current meta-analyses did include a substantial number of fathers, enabling us to gain some insights into sex differences. However, the majority of the studies (almost) exclusively included mothers. The combination of sex-specific results and the overall limited number of studies including fathers, calls for future research that includes fathers and further examines sex (or gender) effects, such as to what extent fathers and mothers differ in their behaviors and how children perceive and react differently to fathers versus mothers. Second, whereas it is important to disentangle the effects of observed and perceived parenting, it is also important to keep in mind that the needs and perception of parents may differ from those of children. It is interesting in this respect that stronger effects are found for perceived than observed parenting with childhood depression, both when looking into questionnaires of perceived parenting (McLeod et al., 2007) as well as when looking into perceived parenting in specific interactions (Wentholt et al., 2025). This means that acknowledgment and alignment of parenting behaviors with the needs of children is of great importance. Lastly and related to the former point, it is important to be aware of parent-blaming. Parenting is not an isolated process; parents react to children and versa, and parents have their own history and sensitivities related to mental health and parents. Moreover, parents and children function within broader systemic and societal circumstances (Bronfenbrenner, 1977; Luijk & Roseboom, 2025). In order to understand, and ultimately prevent and treat childhood depression we therefore need to better understand the underlying processes and triggers for parental criticism or lack of warmth towards a child and develop a broader systemic vision.

### Observed Child Behaviors

In order to yield a more complete picture of the parent–child interactions in the context of childhood depression, the current study further aimed to provide a meta-analytic overview of the observed behaviors of *children* with depression in interactions with their parents. We found sound evidence for increased negative affect, decreased autonomy and task engagement among children with depression. Further, we found limited evidence for decreased positive affect and increased observed depressed affect among children with depression. Increased withdrawal/disengagement was also not consistently observed among children with depressive symptoms. Lastly, based on moderator analyses, we generally found no indications for differences in the relation between observed child behavior and childhood depression based on child age, sex, or the context of the interaction (with mother/father, type of task).

The results of observed child affect partly align with the ‘maladaptive emotion regulation’ in children with depression (suppressed or increased emotional expressions), as reviewed by Chapman et al. (2016). Our results indicate that for children’s *observable* affect states findings are strongest for negative affect, including affect that is characterized by sadness, anxiety and/or irritability, rather than positive affect and depressed affect (more specific, excluding the more externalizing aspects of negative affect). The latter aligns with previous findings that childhood depression is relatively more often characterized by a non-melancholic, irritable/hostile than a melancholic pattern (Crowe et al., 2006; Nardi et al., 2013; Parker & Roy, 2001). Parental responses to their child’s expressed negativity matter, as reciprocation of negativity in families can relate to increased childhood depression (Schwartz et al., 2012a, 2012b). This fits the negative pattern as described in the social interaction theory, for example with child irritability being followed by more parental criticism, which in turn can worsen the depressive state (Coyne, 1976). In addition, research shows that also positive parenting may have a reinforcing effect on negative child behavior, consistent with principles of operant conditioning. For example, increased positive parenting behaviors (and reductions in aggressive behavior) following child dysphoric behavior can lengthen the duration of the child’s dysphoric behavior, and thus function as a reinforcer for child dysphoria (Schwartz et al., 2012a, 2012b). These operant principles may also apply and reinforce children’s negative affect (e.g. irritability). Future research could examine the most optimal parental responses to negative child behaviors and affect, to optimize targets for interventions.

### Moderation of Clinical Status of Childhood Depression

Contrary to our hypothesis, clinical status did not moderate the relation between observed parent–child interaction behaviors and childhood depression, and we thus found no evidence for different effects between studies examining childhood depressive disorder as compared to depressive symptoms. We could not test this hypothesis for all constructs because of an insufficient number of effect sizes for some constructs. Previous meta-analytic research on the relation between parenting and childhood depression, including many studies on perceived and some on observed parenting, showed stronger effects in studies examining depressive disorder than symptoms (McLeod et al., 2007). We did not replicate this in the current study on observed parent–child interaction behaviors and childhood depression.

### Practical Implications

The results of the current study can be translated into three main clinical implications. First, parental lack of warmth and support should be considered as a target for childhood depression prevention in at-risk families and for parenting interventions for parents with children with (sub)clinical depression. For children with depressive symptoms, regardless of sex and age, it is important to assess current parental warmth/support and teach parents on how they can strengthen this behavior. Particularly for younger children with (sub)clinical depression it is important to also consider how parents can take care of themselves in order to be able to maintain parental warmth/support over time, given the findings of warmth/support erosion over time in this subgroup.

Second, parental harsh control and criticism should be considered as a second target for prevention and intervention for childhood depression. It is especially important for parents to limit their harsh control and criticism in interactions with their child in positive, collaborative contexts (e.g., planning an event). Keeping these interactions free of criticism may have a preventive effect for childhood depression. Given the differences for mothers and fathers, it seems important to mainly target harsh control/criticism of mothers in the context of prevention. On the other hand, fathers may respond with increased harsh control/criticism to their child’s depression, at the moment the depression occurs, and possibly also over time. It may be valuable to examine mechanisms that underly this harsh control/criticism (e.g., frustration with the child’s negative behaviors or affect) and ways for parents to deal with this in a more positive manner.

Third, we found sound evidence for increased expressed negative affect and reduced autonomy and task engagement among children with depression in interactions with their parents. Depression in children may be difficult to notice, given the internalizing nature of depression. Increased negative affect (e.g., hostility), reduced autonomy (e.g., difficulty in decision-making), and reduced task engagement (e.g., lowered attention) are more visible and may help to signal depressive feelings and symptoms to parents and professionals. Additionally, these behaviors of children with depression may pose challenges to children as well as parents. Family interventions could validate and address these challenges.

### Strengths and Limitations

The current set of meta-analyses has several strengths. We made a substantial effort to include all relevant studies, resulting in a large number of effect sizes (*K*_*ES*_ = 350 parental behavior, *K*_*ES*_ = 131 child behavior) from 80 unique samples. We used three-level meta-analyses in order to include multiple effect sizes from the same sample (e.g., for mothers and fathers separately). We distinguished between different types of parent and child behaviors, providing insights into the most relevant constructs and highlighting the importance to disentangle constructs with a positive and negative valence (e.g., results for child positive and negative affect varied). Lastly, we ran separate analyses for cross-sectional and longitudinal studies on the relation between observed parenting and childhood depression (in both directions), providing cautious insights into the directionality of this relation.

Naturally, the current study comes with several limitations. First, contrary to our preregistration, we did not assess the relation of conditional (or bidirectional) parent–child interaction behaviors with childhood depression. Examining conditional interactions captures how parents respond (e.g., critical) to specific child behaviors (e.g., hostility) and, vice versa, how children respond (e.g., facilitative/nurturing) to their parent’s behavior or affect (e.g., depressed affect). This may give more detailed insight in the family dynamics and how parents influence children’s emotion regulation style. Given the extensive number of analyses in the current study, we decided this topic should be studied separately. Second, several moderators could not be tested because of an insufficient number of available effect sizes (see Supplementary Table 1). And third, there was a limited number of studies that controlled for baseline measures in testing the longitudinal relation from observed parenting to childhood depression and vice versa as this was not the main aim of many of the studies. Longitudinal associations should therefore be interpreted with some caution, as the current findings may underestimate or overestimate prospective effects.

## Conclusion

To conclude, results of this set of multilevel meta-analyses highlight three key messages. First, observed parental low warmth/support and higher harsh control/criticism co-occur with and precede childhood depression. The previously established link between perceived parenting and depression thus holds when eliminating perception biases on parenting and is mainly observed before and during periods of heightened childhood depression. Childhood depression is generally not *followed* by less positive and more negative observed parenting over time, although this was the case for specific subgroups: Lower warmth was observed in families with young children, and more criticism by fathers after childhood depression. Second, children with depression show more negative affect, reduced autonomy, and reduced task engagement in interactions with their parents. How families can best cope with and regulate these behaviors is an important topic to address in clinical practice. Lastly, we found no evidence for differences between families with a child with increased depressive symptoms as compared to a depressive disorder. In all of this, it is vital to keep in mind that parenting is not an isolated process and that family members can have different perspectives and needs. When targeting parenting to prevent or intervene on childhood depression, a systemic approach means to consider the multiple perspectives and the broader context (school, work, social, family and societal) of the child and parents.

## Supplementary Information

Below is the link to the electronic supplementary material.

Supplementary file1 (DOCX 1727 KB)

## Data Availability

Data and R script will be made available at OSF upon publication.
