# Supplementary material for: Beyond Perceptions: Meta-Analyses on Observed Parent–Child Interaction Behaviors and Childhood and Adolescent Depression
Source: Clin Child Fam Psychol Rev. 2026 Jun 5;29(3):535–70. doi: 10.1007/s10567-026-00572-8 (PMC13562138; doi:10.1007/s10567-026-00572-8)
Supplement: Supplementary file 1 [file 10567_2026_572_MOESM1_ESM.docx]

**Beyond Perceptions: Meta-Analyses on Observed Parent-Child Interaction Behaviors and Childhood and Adolescent Depression**

Manuscript submitted to Clinical Child and Family Psychology Review

Wilma G. M. Wentholt, Marc L. Molendijk, Loes H. C. Janssen, Lisanne A. E. M. van Houtum, Marieke S. Tollenaar, Lenneke R. A. Alink, & Bernet M. Elzinga

**Correspondence.** Wilma G. M. Wentholt, Leiden University, Faculty of Social and Behavioral Sciences, 2333 AK Leiden, the Netherlands. E-mail: [w.g.m.wentholt@fsw.leidenuniv.nl](mailto:w.g.m.wentholt@fsw.leidenuniv.nl). ORCID ID: 0000-0001-9713-3384

**Supplementary Methods**

**Quality Assessment**

The quality of studies was assessed based on seven items, these were summed with a higher score representing higher quality. The items are based on the assessment tool for observational cohort and cross-sectional studies of the National Heart, Lung, and Blood Institute (NHLBI) (National Heart, Lung, and Blood Institute, 2021) and one item of the Newcastle-Ottawa Scale (NOS) (Wells et al., 2021).

1. Population (based on NHLBI): Were all subjects selected/recruited from a similar population? Or in case of two groups; were groups matched based on age and sex?

0 = Not a similar population, not matched, or unclear.

1 = Groups were matched or it was checked whether groups were similar; if groups were not similar authors controlled for variables that differed between groups.

2. Validity assessment depression (based on NHLBI): Was a valid instrument used to assess childhood depression?

0 = Unvalidated instrument, 1-item, clinical assessment without use of a structured instrument.

1 = Validated instrument.

3. Reliability assessment depression (based on NHLBI).
   a. Questionnaire: Was the internal reliability sufficient?

0 = No, Cronbach’s alpha <.60 or in text ‘low reliability’ is reported or authors do not report anything about reliability.

1 = Yes, Cronbach’s alpha >.60 or in text ‘moderate/high reliability’ is reported. This has to concern reliability of the study population, not general reliability or reliability in other studies.

b. Diagnostic interview: Was the inter-rater reliability for scoring of the interview sufficient?

0 = ICC <.70 or kappa <.60 or inter-rater reliability is not reported.

1 = ICC >.70 or kappa >.60.

4. Reliable coders (NHLBI): Was the inter-rater reliability for scoring observed behavior sufficient?

0 = ICC <.70 or kappa <.60 or inter-rater reliability is not reported.

1 = ICC >.70 or kappa >.60.

5. Blinded (based on NHLBI): Were raters of observed behavior blind to group status or other relevant information of the participants?

0 = Unclear or it is reported they were not blind to participant information.

1 = In text it is mentioned raters were blinded or independent or unfamiliar with group and other variables.

6. Missing data (based on NOS): What is the amount of missing data?

0 = >30% of start sample or unknown and cannot be deducted

1 = Missing data <30% of start sample

7. Peer reviewed: Was the study peer reviewed?

0 = Not a peer reviewed journal

1 = Peer reviewed journal

**Effect Size Extraction**

The *escalc* function of the *metafor* package in R (Viechtbauer, 2010) was used to convert mean, standard deviation, sample size per group into standardized mean differences, which was subsequently converted into *r* using the following equation (Borenstein et al., 2009): $r=\frac{d}{\sqrt{d^{2} + 4}}$

We converted β or standardized *b* values of regression analyses to *r* using the *psychometrica* calculator (Lenhard & Lenhard, 2022). If *t* values, but not β values, were reported for univariate regression analyses, the *t* value was converted to *r* using the *escalc* function in R. For nonparametric Wilcoxon rank sum tests, we converted *z* values to Cohen’s *d* using the *psychometrica* calculator (Lenhard & Lenhard, 2022) or converted the median and interquartile range to Cohen’s *d* using the equations by Wan et al. (2014); next, Cohen’s *d* was converted to *r* using the previously mentioned equation. Incidence rates (e.g., sample size of a low v. high behavioral profile for a group of children with depression v. control group) were converted to odds ratios, and odds ratios to *r* using the *psychometrica* calculator (Lenhard & Lenhard, 2022).

We contacted several authors to inquire relevant data or effect sizes. We contacted prof. Bureau to check whether we understood the directions of effects correctly (Bureau et al., 2009). He responded that the observed sensitivity variable was not transformed in the final analyses and a higher score represented more sensitivity, which was requested to be changed in the publication process. Further, he referred us to the footnote in Table 1 of the paper, indicating that a higher score on the depression variable at age 8 years represents *lower* depressive symptoms, whereas a higher score on the variable at age 19 years represents *higher* depressive symptoms. We processed the effect sizes based on this information.

In one case we could not find contact details of the author of a doctoral dissertation nor of researchers mentioned in the acknowledgement (Kulcsar, 1996). Most authors (and/or coauthors) did not respond to our mail and two reminders (after two and four weeks of initial mail) (Bosquet, 2001; Brumariu et al., 2018; Coffino, 2010; Compton, 2000; Cui, 2013; DeGarmo & Forgatch, 2007; Dietz et al., 2014; Fincham et al., 1998; Gonzales, 1992; Human et al., 2016; Hutchinson et al., 2019; Katz & Hunter, 2007; Kerns et al., 2011; Micol, 2023; Milan et al., 2013; Milan & Carlone, 2018; Pozzi et al., 2020; Silver et al., 2023; Stocker et al., 2003; Weaver, 2009). One author actively refused to provide the data (Feinberg et al., 2007). Other (co)authors responded that data were no longer available (Ben-Knaz, 2002; Bosquet, 2001; Crowell et al., 1991; Duggal et al., 2001; Fincham et al., 1998; Franco, 2007; Gonzales, 1992; Gordis et al., 2001; Jarvis, 2006; Lasko et al., 1997; Levy-Shiff et al., 1997; Liebman, 1998; Priel et al., 2019; Schraedley, 2002; Siqueland, 1993; Tarullo et al., 1994; Wilson, 2005) or they had no current resources to set up and send the data (Sheeber et al., 2007; Sulik et al., 2015). Two authors responded that they themselves had no current access to the data and referred us to colleagues, who did not respond to our mails and reminders (Gadassi Polack et al., 2021; Kim Park et al., 2008).

| **Supplementary Table 1**  *Variables Excluded from Moderation Analyses due to an Insufficient Number of Unique Samples* | | | |
| --- | --- | --- | --- |
|  | | *k_samples_* | *k_ES_* |
| **Cross-sectional** | | | |
| Parental autonomy granting | | | |
| Type of task | |  |  |
|  | Problem solving | 1 | 2 |
|  | Event planning | 0 | 0 |
|  | Reminiscence | 0 | 0 |
|  | Teaching | 2 | 7 |
|  | Natural observation | 0 | 0 |
|  | Other | 3 | 4 |
| Informant depression | |  |  |
|  | Child | 5 | 10 |
|  | Parent | 0 | 0 |
|  | Child and parent | 0 | 0 |
|  | Other | 0 | 0 |
| Source assessment depression | |  |  |
|  | Interview | 0 | 0 |
|  | Questionnaire | 5 | 13 |
|  | Other | 0 | 0 |
| Type of comparison | |  |  |
|  | Group-clinical | 0 | 0 |
|  | Group-questionnaire cut-off | 0 | 0 |
|  | Continuous | 5 | 13 |
| Parental guidance/structure | | | |
| Type of task | |  |  |
|  | Problem solving | 2 | 2 |
|  | Event planning | 0 | 0 |
|  | Reminiscence | 0 | 0 |
|  | Teaching | 3 | 4 |
|  | Natural observation | 0 | 0 |
|  | Other | 1 | 1 |
| Informant depression | |  |  |
|  | Child | 5 | 6 |
|  | Parent | 1 | 1 |
|  | Child and parent | 0 | 0 |
|  | Other | 0 | 0 |
| Source assessment depression | |  |  |
|  | Interview | 2 | 2 |
|  | Questionnaire | 3 | 4 |
|  | Other | 1 | 1 |
| Type of comparison | |  |  |
|  | Group-clinical | 2 | 2 |
|  | Group-questionnaire cut-off | 0 | 0 |
|  | Continuous | 4 | 5 |
| Parental depressed affect | | | |
| Type of task | |  |  |
|  | Problem solving | 3 | 6 |
|  | Event planning | 0 | 0 |
|  | Reminiscence | 0 | 0 |
|  | Teaching | 0 | 0 |
|  | Natural observation | 0 | 0 |
|  | Other | 2 | 7 |
| Informant depression | |  |  |
|  | Child | 5 | 13 |
|  | Parent | 0 | 0 |
|  | Child and parent | 0 | 0 |
|  | Other | 0 | 0 |
| Source assessment depression | |  |  |
|  | Interview | 2 | 7 |
|  | Questionnaire | 2 | 5 |
|  | Other | 1 | 1 |
| Type of comparison | |  |  |
|  | Group-clinical | 3 | 8 |
|  | Group-questionnaire cut-off | 0 | 0 |
|  | Continuous | 2 | 5 |
| Child depressed affect | | | |
| Type of task | |  |  |
|  | Problem solving | 5 | 8 |
|  | Event planning | 1 | 3 |
|  | Reminiscence | 0 | 0 |
|  | Teaching | 0 | 0 |
|  | Natural observation | 0 | 0 |
|  | Other | 1 | 1 |
| Informant depression | |  |  |
|  | Child | 6 | 12 |
|  | Parent | 0 | 0 |
|  | Child and parent | 0 | 0 |
|  | Other | 0 | 0 |
| Source assessment depression | |  |  |
|  | Interview | 3 | 4 |
|  | Questionnaire | 2 | 7 |
|  | Other | 1 | 1 |
| Type of comparison | |  |  |
|  | Group-clinical | 4 | 5 |
|  | Group-questionnaire cut-off | 0 | 0 |
|  | Continuous | 2 | 7 |
| Child autonomy | | | |
| Type of task | |  |  |
|  | Problem solving | 5 | 14 |
|  | Event planning | 0 | 0 |
|  | Reminiscence | 0 | 0 |
|  | Teaching | 0 | 0 |
|  | Natural observation | 0 | 0 |
|  | Other | 2 | 2 |
| Informant depression | |  |  |
|  | Child | 5 | 10 |
|  | Parent | 0 | 0 |
|  | Child and parent | 1 | 2 |
|  | Other | 1 | 4 |
| Source assessment depression | |  |  |
|  | Interview | 1 | 2 |
|  | Questionnaire | 5 | 10 |
|  | Other | 1 | 4 |
| Type of comparison | |  |  |
|  | Group-clinical | 1 | 2 |
|  | Group-questionnaire cut-off | 1 | 4 |
|  | Continuous | 5 | 10 |
| Child task engagement | | | |
| Informant depression | |  |  |
|  | Child | 8 | 11 |
|  | Parent | 2 | 6 |
|  | Child and parent | 1 | 1 |
|  | Other | 1 | 6 |
| Child withdrawal/unengaged | | | |
| Type of task | |  |  |
|  | Problem solving | 3 | 4 |
|  | Event planning | 0 | 0 |
|  | Reminiscence | 0 | 0 |
|  | Teaching | 1 | 1 |
|  | Natural observation | 0 | 0 |
|  | Other | 1 | 1 |
| Informant depression | |  |  |
|  | Child | 2 | 2 |
|  | Parent | 1 | 1 |
|  | Child and parent | 2 | 3 |
|  | Other | 0 | 0 |
| Source assessment depression | |  |  |
|  | Interview | 3 | 4 |
|  | Questionnaire | 2 | 2 |
|  | Other | 0 | 0 |
| Type of comparison | |  |  |
|  | Group-clinical | 2 | 2 |
|  | Group-questionnaire cut-off | 0 | 0 |
|  | Continuous | 3 | 4 |
| **Longitudinal** | | | |
| Parental warmth/support 🡪 childhood depression | | | |
| Informant depression | |  |  |
|  | Child | 15 | 54 |
|  | Parent | 2 | 2 |
|  | Child and parent | 2 | 4 |
|  | Other | 0 | 0 |
| Type of comparison | |  |  |
|  | Group-clinical | 2 | 4 |
|  | Group-questionnaire cut-off | 0 | 0 |
|  | Continuous | 17 | 57 |
| Childhood depression 🡪 parental warmth/support | | | |
| Type of task | |  |  |
|  | Problem solving | 2 | 4 |
|  | Event planning | 0 | 0 |
|  | Reminiscence | 0 | 0 |
|  | Teaching | 0 | 0 |
|  | Natural observation | 0 | 0 |
|  | Other | 2 | 3 |
| Informant depression | |  |  |
|  | Child | 3 | 5 |
|  | Parent | 2 | 2 |
|  | Child and parent | 0 | 0 |
|  | Other | 0 | 0 |
| Source assessment depression | |  |  |
|  | Interview | 0 | 0 |
|  | Questionnaire | 3 | 5 |
|  | Other | 1 | 2 |
| Type of comparison | |  |  |
|  | Group-clinical | 0 | 0 |
|  | Group-questionnaire cut-off | 0 | 0 |
|  | Continuous | 4 | 7 |
| Parental harsh control/criticism 🡪 childhood depression | | | |
| Type of comparison | |  |  |
|  | Group-clinical | 2 | 3 |
|  | Group-questionnaire cut-off | 0 | 0 |
|  | Continuous | 16 | 51 |
| Childhood depression 🡪 parental harsh control/criticism | | | |
| Age children | | 2 | 3 |
| Type of task | |  |  |
|  | Problem solving | 2 | 4 |
|  | Event planning | 0 | 0 |
|  | Reminiscence | 0 | 0 |
|  | Teaching | 0 | 0 |
|  | Natural observation | 0 | 0 |
|  | Other | 1 | 1 |
| Informant depression | |  |  |
|  | Child | 2 | 3 |
|  | Parent | 2 | 2 |
|  | Child and parent | 0 | 0 |
|  | Other | 0 | 0 |
| Source assessment depression | |  |  |
|  | Interview | 0 | 0 |
|  | Questionnaire | 2 | 3 |
|  | Other | 1 | 2 |
| Type of comparison | |  |  |
|  | Group-clinical | 0 | 0 |
|  | Group-questionnaire cut-off | 0 | 0 |
|  | Continuous | 3 | 5 |
| *Note.* Continuous moderators: minimally three unique samples should be available for analyses. Categorical moderators: minimally three unique samples for minimally two categories should be available for analyses. | | | |

**Supplementary Figure 1**

*Funnel and Forest Plot of the Cross-Sectional Relation of Parental Warmth/Support and Childhood Depression*

**Supplementary Figure 2**

*Funnel and Forest Plot of the Longitudinal Relation of Parental Warmth/Support (T1) and Childhood Depression (T2)*

**Supplementary Figure 3**

*Funnel and Forest Plot of the Longitudinal Relation of Childhood Depression (T1) and Parental Warmth/Support (T2)*

**Supplementary Figure 4**

*Funnel and Forest Plot of the Cross-Sectional Relation of Parental Harsh Control/Criticism and Childhood Depression*

**Supplementary Figure 5**

*Funnel and Forest Plot of the Longitudinal Relation of Parental Harsh Control/Criticism (T1) and Childhood Depression (T2)*

**Supplementary Figure 6**

*Funnel and Forest Plot of the Longitudinal Relation of Childhood Depression (T1) and Parental Harsh Control/Criticism (T2)*

**Supplementary Figure 7**

*Funnel and Forest Plot of the Cross-Sectional Relation of Parental Autonomy Granting and Childhood Depression*

**Supplementary Figure 8**

*Funnel and Forest Plot of the Cross-Sectional Relation of Parental Guidance/Structure and Childhood Depression*

**Supplementary Figure 9**

*Funnel and Forest Plot of the Cross-Sectional Relation of Parental Depressed Affect and Childhood Depression*

**Supplementary Figure 10**

*Funnel and Forest Plot of the Cross-Sectional Relation of Child Positive Affect and Childhood Depression*

**Supplementary Figure 11**

*Funnel and Forest Plot of the Cross-Sectional Relation of Child Negative Affect and Childhood Depression*

**Supplementary Figure 12**

*Funnel and Forest Plot of the Cross-Sectional Relation of Child Depressed Affect and Childhood Depression*

**Supplementary Figure 13**

*Funnel and Forest Plot of the Cross-Sectional Relation of Child Autonomy and Childhood Depression*

**Supplementary Figure 14**

*Funnel and Forest Plot of the Cross-Sectional Relation of Child Task Engagement and Childhood Depression*

**Supplementary Figure 15**

*Funnel and Forest Plot of the Cross-Sectional Relation of Child Withdrawal/Unengaged and Childhood Depression*

**References**

Ben-Knaz, R. (2002). *Family interaction in differentially diagnosed academically-able male adolescent underachievers* [Doctoral Dissertation, York University]. ProQuest Doctoral Dissertations and Theses.

Borenstein, M., Hedges, L. V., Higgins, J. P. T., & Rothstein, H. R. (2009). *Introduction to meta-analysis.* New Jersey, NJ: John Wiley & Sons.

Bosquet, M. A. (2001). *An examination of the development of anxiety symptoms from a developmental psychopathology perspective* [Doctoral Dissertation, University of Minnesota]. ProQuest Doctoral Dissertations and Theses.

Brumariu, L. E., Giuseppone, K. R., Kerns, K. A., Van de Walle, M., Bureau, J.-F., Bosmans, G., & Lyons-Ruth, K. (2018). Middle childhood attachment strategies: Validation of an observational measure. *Attachment and Human Development, 20*(5), 491–513. https://doi.org/10.1080/14616734.2018.1433696

Bureau, J.-F., Easterbrooks, M. A., & Lyons-Ruth, K. (2009). Maternal depressive symptoms in infancy: Unique contribution to children’s depressive symptoms in childhood and adolescence? *Development and Psychopathology, 21*(2), 519–537. https://doi.org/10.1017/S0954579409000285

Coffino, B. S. (2010). *The role of depression and social relationships in the intergenerational transmission of observed parenting* [Doctoral Dissertation, University of Minnesota]. ProQuest Doctoral Dissertations and Theses.

Compton, K. L. (2000). *A double jeopardy model of risk: The additive effects of maternal depression and sibling psychopathology in negative child outcome by gender* [Doctoral Dissertation, Wichita State University]. ProQuest Doctoral Dissertations and Theses.

Crowell, J. A., O’Connor, E., Wollmers, G., Sprafkin, J., & Rao, U. (1991). Mothers’ conceptualizations of parent-child relationships: Relation to mother-child interaction and child behavior problems. *Development and Psychopathology, 3*(4), 431–444. https://doi.org/10.1017/S0954579400007616

Cui, L. (2013). *Respiratory sinus arrhythmia baseline and reactivity to interpersonal challenge: A dynamic systems approach* [Doctoral Dissertation, Oklahoma State University]. ProQuest Doctoral Dissertations and Theses.

DeGarmo, D. S., & Forgatch, M. S. (2007). Efficacy of parent training for stepfathers: From playful spectator and polite stranger to effective stepfathering. *Parenting: Science and Practice, 7*(4), 331–355. https://doi.org/10.1080/15295190701665631

Dietz, L. J., Marshal, M. P., Burton, C. M., Bridge, J. A., Birmaher, B., Kolko, D., Duffy, J. N., & Brent, D. A. (2014). Social problem solving among depressed adolescents is enhanced by structured psychotherapies. *Journal of Consulting and Clinical Psychology, 82*(2), 202.

Duggal, S., Carlson, E. A., Sroufe, L. A., & Egeland, B. (2001). Depressive symptomatology in childhood and adolescence. *Development and Psychopathology, 13*(1), 143–164. https://doi.org/10.1017/S0954579401001109

Feinberg, M. E., Button, T. M., Neiderhiser, J. M., Reiss, D., & Hetherington, E. M. (2007). Parenting and adolescent antisocial behavior and depression: Evidence of genotype\times parenting environment interaction. *Archives of General Psychiatry, 64*(4), 457–465.

Fincham, F. D., Beach, S. R. H., Arias, I., & Brody, G. H. (1998). Children’s attributions in the family: The Children’s Relationship Attribution Measure. *Journal of Family Psychology, 12*(4), 481–493. https://doi.org/10.1037/0893-3200.12.4.481

Franco, X. (2007). *Evaluating the external validity of comorbid disorders in youth using rating scales and behavior observations* [Doctoral Dissertation, Florida International University]. ProQuest Doctoral Dissertations and Theses.

Gonzales, N. A. (1992). *Parent-adolescent relations and adolescent adjustment in African-American families* [Doctoral Dissertation, University of Washington]. ProQuest Doctoral Dissertations and Theses.

Gordis, E. B., Margolin, G., & John, R. S. (2001). Parents’ hostility in dyadic marital and triadic family settings and children’s behavior problems. *Journal of Consulting and Clinical Psychology, 69*(4), 727–734. https://doi.org/10.1037/0022-006X.69.4.727

Human, L. J., Chan, M., Ifthikhar, R., Williams, D., DeLongis, A., & Chen, E. (2016). Accuracy and positivity in adolescent perceptions of parent behavior: Links with adolescent psychological adjustment and proinflammatory profiles. *Social Psychological and Personality Science, 7*(8), 796–805. https://doi.org/10.1177/1948550616660590

Hutchinson, E. A., Rosen, D., Allen, K., Price, R. B., Amole, M., & Silk, J. S. (2019). Adolescent gaze-directed attention during parent–child conflict: The effects of depressive symptoms and parent–child relationship quality. *Child Psychiatry and Human Development, 50*(3), 483–493. https://doi.org/10.1007/s10578-018-0856-y

Jarvis, K. L. (2006). *Maternal parenting and child adjustment in the context of domestic violence* [Doctoral Dissertation, University of California, Irvine]. ProQuest Doctoral Dissertations and Theses.

Katz, L. F., & Hunter, E. C. (2007). Maternal meta-emotion philosophy and adolescent depressive symptomatology. *Social Development, 16*(2), 343–360. https://doi.org/10.1111/j.1467-9507.2007.00388.x

Kerns, K. A., Brumariu, L. E., & Seibert, A. (2011). Multi-method assessment of mother-child attachment: Links to parenting and child depressive symptoms in middle childhood. *Attachment and Human Development, 13*(4), 315–333. https://doi.org/10.1080/14616734.2011.584398

Kim Park, I. J., Garber, J., Ciesla, J. A., & Ellis, B. J. (2008). Convergence among multiple methods of measuring positivity and negativity in the family environment: Relation to depression in mothers and their children. *Journal of Family Psychology, 22*(1), 123–134. https://doi.org/10.1037/0893-3200.22.1.123

Kulcsar, E. N. (1996). *Maternal depression and separation-individuation in adolescent daughters* [Doctoral Dissertation, The University of Utah]. ProQuest Doctoral Dissertations and Theses.

Lasko, D., Field, T., Bendell, D., Yando, R., Scafidi, F., LaGreca, A., & Trapani, L. (1997). Adolescent psychiatric patients’ interactions with their mothers. *Adolescence, 32*(128), 977–988.

Lenhard, W., & Lenhard, A. (2022). *Computation of effect Sizes* [Dataset]. Unpublished. https://doi.org/10.13140/RG.2.2.17823.92329

Levy-Shiff, R., Zoran, N., & Shulman, S. (1997). International and domestic adoption: Child, parents, and family adjustment. *International Journal of Behavioral Development, 20*(1), 109–129. https://doi.org/10.1080/016502597385478

Liebman, S. E. (1998). *Familial predictors of depressed mood in adolescence* [Doctoral Dissertation, University of Virginia]. ProQuest Doctoral Dissertations and Theses.

Micol, V. J. (2023). *Parenting and family-related protective factors for adolescents at high risk for depression and suicide* [Doctoral Dissertation, University of Michigan]. ProQuest Doctoral Dissertations and Theses.

Milan, S., & Carlone, C. (2018). A two-way street: Mothers’ and adolescent daughters’ depression and PTSD symptoms jointly predict dyadic behaviors. *Journal of Family Psychology, 32*(8), 1097–1108. https://doi.org/10.1037/fam0000467

Milan, S., Zona, K., & Snow, S. (2013). Pathways to adolescent internalizing: Early attachment insecurity as a lasting source of vulnerability. *Journal of Clinical Child and Adolescent Psychology, 42*(3), 371–383. https://doi.org/10.1080/15374416.2012.736357

National Heart, Lung, and Blood Institute. (2021). *Study quality assessment tools*. https://www.nhlbi.nih.gov/health-topics/study-quality-assessment-tools

Polack, R. G., Joormann, J., Orbach, M., Silverman, W. K., & Lebowitz, E. R. (2021). Maternal depression and mother-child oxytocin synchrony in youth with anxiety disorders. *Research on Child and Adolescent Psychopathology, 49*(3), 381–392. https://doi.org/10.1007/s10802-020-00744-2

Pozzi, E., Simmons, J. G., Bousman, C. A., Vijayakumar, N., Bray, K. O., Dandash, O., Richmond, S., Schwartz, O., Seal, M., Sheeber, L., Yap, M. B. H., Allen, N. B., & Whittle, S. L. (2020). The influence of maternal parenting style on the neural correlates of emotion processing in children. *Journal of the American Academy of Child and Adolescent Psychiatry, 59*(2), 274–282. https://doi.org/10.1016/j.jaac.2019.01.018

Priel, A., Djalovski, A., Zagoory-Sharon, O., & Feldman, R. (2019). Maternal depression impacts child psychopathology across the first decade of life: Oxytocin and synchrony as markers of resilience. *Journal of Child Psychology and Psychiatry, 60*(1), 30–42. https://doi.org/10.1111/jcpp.12880

Schraedley, P. K. (2002). *Intergenerational transmission of psychopathology: Psychosocial difficulties among daughters of depressed mothers* [Doctoral Dissertation, Stanford University]. ProQuest Doctoral Dissertations and Theses.

Sheeber, L. B., Davis, B., Leve, C., Hops, H., & Tildesley, E. (2007). Adolescents’ relationships with their mothers and fathers: Associations with depressive disorder and subdiagnostic symptomatology. *Journal of Abnormal Psychology, 116*(1), 144–154. https://doi.org/10.1037/0021-843X.116.1.144

Silver, J., Bufferd, S. J., Dougherty, L. R., Goldstein, B. L., Carlson, G. A., & Klein, D. N. (2023). Is the distinction between tonic and phasic irritability meaningful in 3-year-old children? *European Child and Adolescent Psychiatry, 32*(9), 1755–1763. https://doi.org/10.1007/s00787-022-01995-8

Siqueland, L. R. (1993). Anxiety in children: Perceived family environments and observed family interaction styles [Doctoral Dissertation, Temple University]. ProQuest Doctoral Dissertations and Theses.

Stocker, C. M., Richmond, M. K., Low, S. M., Alexander, E. K., & Elias, N. M. (2003). Marital conflict and children’s adjustment: Parental hostility and children’s interpretations as mediators. *Social Development, 12*(2), 149–161. https://doi.org/10.1111/1467-9507.00226

Sulik, M. J., Eisenberg, N., Spinrad, T. L., Lemery-Chalfant, K., Swann, G., Silva, K. M., Reiser, M., Stover, D. A., & Verrelli, B. C. (2015). Interactions among catechol-O-methyltransferase genotype, parenting, and sex predict children’s internalizing symptoms and inhibitory control: Evidence for differential susceptibility. *Development and Psychopathology, 27*(3), 709–723. https://doi.org/10.1017/S0954579414000807

Tarullo, L. B., DeMulder, E. K., Martinez, P. E., & Radke-Yarrow, M. (1994). Dialogues with preadolescents and adolescents: Mother-child interaction patterns in affectively ill and well dyads. *Journal of Abnormal Child Psychology, 22*(1), 33–51. https://doi.org/10.1007/BF02169255

Viechtbauer, W. (2010). Conducting meta-analyses in R with the metafor package. *Journal of Statistical Software, 36*(3), 1–48. https://doi.org/10.18637/jss.v036.i03

Wan, X., Wang, W., Liu, J., & Tong, T. (2014). Estimating the sample mean and standard deviation from the sample size, median, range and/or interquartile range. *BMC Medical Research Methodology, 14*(1), 135. https://doi.org/10.1186/1471-2288-14-135

Weaver, J. M. (2009). *Setting the tone: Understanding how the family context transmits risks for adolescents of parents with depressive symptoms and high levels of anger* [Doctoral Dissertation, University of California, Irvine]. ProQuest Doctoral Dissertations and Theses.

Wells, G. A., O’Connell, D., Peterson, J., Welch, V., Losos, M., & Tugwell, P. (2021). *The Newcastle-Ottowa Scale for assessing the quality of nonrandomised studies in meta-analyses.* https://www.ohri.ca/programs/clinical_epidemiology/oxford.asp

Wilson, H. W. (2005). *Parent-teen communication about sex: Links to partner negotiation attitudes and risky sexual behavior among adolescents in psychiatric treatment* [Doctoral Dissertation, Northwestern University]. ProQuest Doctoral Dissertations and Theses.
